# Supplementary material for: Income inequality and social gradients in children’s height: a comparison of cohort studies from five high-income countries
Source: BMJ Paediatr Open. 2019 Nov 28;3(1):e000568. doi: 10.1136/bmjpo-2019-000568 (PMC6937032; doi:10.1136/bmjpo-2019-000568)
Supplement: Supplementary data [file bmjpo-2019-000568supp001.pdf]

Income inequality and social gradients in children's height: a comparison of cohort studies from five high-income countries

Supplementary file

Supplementary Table 1a: Parental education response categories in each cohort and recoding strategies

Harmonised parental education	ECLS-K (US)	MCS (UK)	LSAC-K (Australia)
Level 1 (Below secondary school qualification/ not completed school)	8th grade or below<br>9th-12th grade	Academic qualification either of: CSE below grade 1/GCSE or O Level below grade C, SCE Standard, Ordinary grades below grade 3, or Junior Certificate below grade C<br>Or vocational qualification any of: NVQ or SVQ Level 1/GNVQ Foundation Level or GSVQ Level 1, BTEC, SCOTVEC first or general certificate/SCOTVEC modules, City & Guilds part 1/RSA Stage I,II,III/Junior certificate	Years of schooling <=10 AND highest qualification = has no certificate
Level 2 (Completed secondary school qualification)	High school diploma/ equivalent	Academic qualification either of: A/AS/S Levels/SCE Higher, Scottish Certificate Sixth Year Studies, Leaving Certificate or equivalent, Level or GCSE grade A-C, SCE Standard, Ordinary grades 1-3, or Junior Certificate grade A-C<br>Or vocational qualification any of: NVQ or SVQ Level 3/GNVQ Advanced or GSVQ Level 3, OND, ONCM BTEC National, SCOTVEC National Certificate, City & Guilds advanced craft, Part III/RSA Advanced Diploma, NVQ or SVQ Level 2/GNVQ Intermediate or GSVQ Level 2, BTEC, SCOTVEC first or general diploma, City & Guilds Craft or Part II/RSA Diploma	Years of schooling >=10 AND highest qualification = has a certificate
Level 3 (Post-secondary or technical qualification)	Vocational/ technical programme<br>Some college<br>Graduate/ professional school – no degree	Academic qualification either of: Post-graduate Diplomas and Certificates, Diplomas in higher education and other higher education qualifications, Teaching qualifications for schools or further education (below degree level)<br>Or vocational qualification any of: Nursing or other medical qualifications (below degree level), NVQ or SVQ level 4 or 5, HND, HNC, Higher Level BTEC/RSA Higher Diploma	Has trade qualification or highest qualification = advanced diploma/diploma
Level 4 (Degree or higher qualification)	Bachelor's degree<br>Masters degree<br>Doctorate/professional degree	Academic qualification either of: Higher Degree and Postgraduate qualifications, First Degree (including B.Ed.)<br>Or vocational qualification any of: Professional qualifications at degree level e.g. graduate member of professional institute, chartered	Highest qualification = bachelor degree or graduate diploma/certificate or postgraduate degree

Bird PK, *et al. BMJ Paediatrics Open* 2019; 3:e000568. doi: 10.1136/bmjpo-2019-000568

		accountant or surveyor	

Supplementary Table 1b: Parental education response categories in each cohort and recoding strategies

Harmonised parental education variable	GenerationR (Netherlands)	ABIS (Sweden)
Level 1(Completed secondary school qualification)	No primary school completed<br>Primary school completed (elementary education)<br>Special/remedial primary education (ZMOK, MLK, ZMLK, BLO, LOM)<br>Special secondary education (VSO-LOM, VSO-MLK)<br>Pre-vocational education (VBO)	Grundskola (elementary/ secondary school)
Level 2 (Lower technical/vocational qualification)	Lower vocational education / secondary general education (VMBO, MAVO, MULO)<br>General secondary education (HAVO, VWO, HBS, MMS, lyceum, atheneum, gymnasium)	Gymnasium, praktisk linje (senior high school – practical)
Level 3 (Higher technical/theoretical qualification)	Senior secondary vocational education (MBO, apprenticeship training)<br>Higher professional education (HBO)	Gymnasium, teoretisk linje (senior high school – theoretical)<br>Folkhögskola (residential college)
Level 4 (Degree or higher qualification)	University degree (WO)	Högskolautbildning högskole/univ-utbildning (college/university)

Supplementary Table 2a: Unadjusted gradients in child height by highest parental education level

	ECLS-K (US)		MCS (UK)		LSAC-K (Australia)		GenerationR (Netherlands)		ABIS (Sweden)	
	n	mean	n	mean	n	mean	n	mean	n	mean
**Girls**										
Level 1 (lowest)	127	111.7	876	109.1	147	107.6	74	118.2	58	112.5
Level 2	974	112.6	2498	109.9	502	107.7	371	118.4	606	113.5
Level 3	1721	113.1	660	110.0	663	108.1	456	118.4	1053	113.3
Level 4 (highest)	1697	113.3	1912	110.7	741	108.4	869	118.7	1585	113.8
Total	4519	113.0	5946	110.1	2053	108.0	1770	118.5	3302	113.6
**Boys**										
Level 1 (lowest)	159	113.4	873	110.0	129	108.6	94	119.7	77	113.5
Level 2	1042	113.6	2688	110.7	525	108.9	367	119.3	705	114.4
Level 3	1720	114.1	683	111.2	705	108.9	451	119.0	1154	114.5
Level 4 (highest)	1843	114.6	1992	111.8	779	109.3	830	119.3	1726	114.6
Total	4764	114.2	6236	111.0	2138	109.0	1742	119.2	3662	114.5

Note: Numbers are unweighted, means are weighted (unweighted in GenerationR and ABIS).

Bird PK, *et al. BMJ Paediatrics Open* 2019; 3:e000568. doi: 10.1136/bmjpo-2019-000568

Supplementary Table 2b: Unadjusted gradients in child height by equivalised household income quintile

	ECLS-K (US)		MCS (UK)		LSAC-K (Australia)		GenerationR (Netherlands)		ABIS (Sweden)	
**Household income quintile**	n	mean	n	mean	n	mean	n	mean	n	mean
**Girls**										
1 (lowest)	836	112.4	1261	109.4	358	107.3	317	118.4	625	113.0
2	883	112.9	1259	109.6	386	107.6	340	118.6	658	113.5
3	905	113.4	1181	110.1	386	108.0	327	119.0	680	113.3
4	942	113.2	1150	110.3	402	108.7	342	118.2	680	113.5
5 (highest)	953	113.2	1089	110.8	462	108.5	334	118.7	665	114.3
Total	4519	113.0	5940	110.1	1994	108.0	1660	118.6	3308	113.6
**Boys**										
1 (lowest)	845	113.4	1296	110.2	380	108.5	345	119.2	762	114.2
2	947	114.2	1324	110.6	401	108.6	322	118.9	740	114.4
3	959	114.3	1268	111.0	430	108.9	336	119.2	716	114.6
4	1000	114.3	1213	111.4	427	109.1	321	119.4	712	114.4
5 (highest)	1013	114.6	1129	111.8	441	109.8	327	119.4	724	114.9
Total	4764	114.2	6230	111.0	2079	109.0	1651	119.2	3654	114.5

Note: Numbers are unweighted, means are weighted (unweighted in GenerationR).

Bird PK, *et al. BMJ Paediatrics Open* 2019; 3:e000568. doi: 10.1136/bmjpo-2019-000568
