# Supplementary material for: Income inequality and social gradients in children’s height: a comparison of cohort studies from five high-income countries
Source: BMJ Paediatr Open. 2019 Nov 28;3(1):e000568. doi: 10.1136/bmjpo-2019-000568 (PMC6937032; doi:10.1136/bmjpo-2019-000568)
Supplement: Author's manuscript [file bmjpo-2019-000568.draft_revisions.pdf]

BMJ Paediatrics Open

BMJ Paediatrics Open is committed to open peer review. As part of this commitment we make the peer review history of every article we publish publicly available.

When an article is published we post the peer reviewers' comments and the authors' responses online. We also post the versions of the paper that were used during peer review. These are the versions that the peer review comments apply to.

The versions of the paper that follow are the versions that were submitted during the peer review process. They are not the versions of record or the final published versions. They should not be cited or distributed as the published version of this manuscript.

BMJ Paediatrics Open is an open access journal and the full, final, typeset and author-corrected version of record of the manuscript is available on our site with no access controls, subscription charges or pay-per-view fees (http://bmjpaedsopen.bmj.com).

If you have any questions on BMJ Paediatrics Open's open peer review process please email info.bmjpo@bmj.com

BMJ Paediatrics Open

Income inequality and social gradients in children's height: a comparison of cohort studies from five high-income countries

Journal:	*BMJ Paediatrics Open*
Manuscript ID	bmjpo-2019-000568
Article Type:	Original article
Date Submitted by the Author:	16-Aug-2019
Complete List of Authors:	Bird, Philippa; Bradford Institute for Health Research, ; University of York, Department of Health Sciences<br>Pickett, Kate; University of York, Health Sciences<br>Graham, Hilary; University of York, Department of Health Sciences<br>Faresjö, Tomas; Linkopings universitet Institutionen for medicin och halsa, Allmänmedicin<br>Jaddoe, Vincent; Erasmus Medical Center, Generation R Study Group; Erasmus Medical Center, Department of Epidemiology<br>Ludvigsson, Johnny; Div of Pediatrics, Dept of Clin Exp Medicine, Medical Faculty, Linköping University, ; Crown Princess Victoria Children´s Hospital, Region Östergötland<br>Raat, Hein; Erasmus Medical Center, Department of Public Health<br>Seguin, Louise; Universite de Montreal, Department of Social and Preventive Medicine<br>Wijtzes, Anne; Erasmus Medical Center, Department of Public Health<br>McGrath, Jennifer; Concordia University, Department of Psychology
Keywords:	Epidemiology, Growth

SCHOLARONE™
Manuscripts

Income inequality and social gradients in children's height: a comparison of cohort studies from five high-income countries

Philippa K. Bird (PhD), Bradford Institute for Health Research, Bradford, UK; Department of Health Sciences, University of York, York, UK

Kate E. Pickett (PhD), Department of Health Sciences, University of York, York, UK

Hilary Graham (PhD), Department of Health Sciences, University of York, York, UK

Tomas Faresjö (PhD), Department of Medical and Health Sciences, Linköping University, Linköping, Sweden

Vincent W.V. Jaddoe (MD), Generation R Study Group, Erasmus Medical Center, Rotterdam, The Netherlands; Department of Epidemiology, Erasmus Medical Center, Rotterdam, The Netherlands; Department of Pediatrics, Erasmus Medical Center, Rotterdam, The Netherlands

Johnny Ludvigsson (PhD), Division of Pediatrics, Department of Clinical and Experimental Medicine, Linköping University, Linköping, Sweden

Hein Raat (MD), Department of Public Health, Erasmus Medical Center, Rotterdam, The Netherlands

Louise Seguin (MD), Department of Social and Preventive Medicine, Université de Montréal, Québec, Canada

Anne I. Wijtzes (PhD), Department of Public Health, Erasmus Medical Center, Rotterdam, the Netherlands

Jennifer J. McGrath (PhD), Pediatric Public Health Psychology Laboratory, Department of Psychology, Concordia University, Montreal, Quebec, Canada

Corresponding author:

Dr Philippa K. Bird

philippa.bird@nhs.net

07811 786851

37 Rhodes Street, Shipley, Bradford, BD18 3JJ

Word count: 2609

Confidential: For Review Only

Abstract

Background

Health and wellbeing are better, on average, in countries that are more equal but less is known about how this benefit is distributed across society. Height is a widely used, objective indicator of child health and predictor of lifelong wellbeing. We compared the level and slope of social gradients in children's height in high-income countries with different levels of income inequality, in order to investigate whether children growing up in all socioeconomic circumstances are healthier in more equal countries.

Methods

We conducted a coordinated analysis of data from five cohort studies from countries that were selected to represent different levels of income inequality (the US, UK, Canada, Australia, the Netherlands and Sweden). We used standardised methods to compare social gradients in children's height at age 4-6 years, by parent education status and household income. We used linear regression models and predicted height for children with the same age, sex and socioeconomic circumstances in each cohort.

Results

The total analytic sample was 37,063 children aged 4-6 years. Gradients by parent education and household income varied between cohorts and outcomes. After adjusting for differences in age and sex, children in more equal countries were taller at all levels of parent education and household income than children in less equal countries, with the greatest between-country differences among children with less educated parents and lowest household incomes.

Conclusions

The study provides preliminary evidence that children across society do better in more equal countries, with greatest benefit among children from the most disadvantaged socioeconomic groups.

Keywords

Income inequality, socioeconomic position, child health, health inequality, cohort study

Confidential: For Review Only

Income inequality and social gradients in child height:

a coordinated analysis of cohort studies from five high-income countries

What is known about the subject

- Countries with lower income inequality have better average health and wellbeing
- There is a social gradient in child health within countries
- There is some limited and inconsistent evidence that health is worse at all points of the social gradient in more unequal countries

What this study adds

- There is considerable variation in the social gap in child height between countries, suggesting that inequalities are not inevitable
- Children are shorter at all points of the social gradient in more unequal societies, with the greatest detriment among children from the most disadvantaged backgrounds

For Review Only

Introduction

On average, countries that are more equal have better health and wellbeing.[1 2] Comparisons of population data have shown that high-income countries with less income inequality have longer life expectancy, lower teenage pregnancy rates, lower infant mortality and better child wellbeing.[3-8] Within countries, the social gradient in health is well-established: each incremental improvement in socioeconomic circumstances brings an associated health gain.[9]

It remains unclear, however, how the health benefit of living in a more equal country is distributed across society - in other words, whether or not both rich and poor do better. One previous ecological study using aggregated mortality data for rich and poor counties within US states found that mortality rates for some causes of death were lower for everyone in more equal US states, but for causes of death with no social gradient, there was no inequality effect.[10] Several studies have used individual data to compare social gradients in height in different countries.[11-17] When they are interpreted in relation to income inequality, they provide some indication that health is better among people at all points in the social hierarchy in more equal countries. For example, a comparative study showed that infant mortality rates were higher overall and the social gradient was steeper in (less equal) England and Wales than (more equal) Sweden. Infant mortality was therefore higher in all social classes in England and Wales than in Sweden, with the greatest difference among lower social class groups.[11] However, there are inconsistencies between studies.

Understanding whether people in all socioeconomic circumstances do better in more equal countries might offer important potential policy levers to address inequality. When considering *child* health, and health-related indicators of lifelong health and wellbeing that can be measured in children, the policy implications are significant. If children's trajectories of health and wellbeing are shaped by both family socioeconomic circumstances and societal levels of inequality, then promoting more equitable health and life chances is a matter of social justice.

Child height is a well-recognised marker of children's social and environmental conditions and general population health. Economic, nutritional and health constraints early in life, as well as psychological and social stressors such as family conflict, reduce children's likelihood of achieving their genetic height potential.[18] Height not only reflects past and current socioeconomic conditions, but is also a good indicator of future wellbeing and success, including better health and workplace success and higher average income.[19 20-22] Within countries, numerous studies have described social gradients in growth and height from birth, through childhood and into adulthood.[23 24] However, if children from affluent families are shorter in more unequal countries than equally well-off families in more equal countries, then the need for policies aimed at inequality reduction (as well as poverty alleviation) might be brought into focus.

This study aimed to answer the question: how do social gradients in child height vary in relation to income inequality in high income countries? We used child height as a marker of current and future health and wellbeing to investigate whether children growing up in all socioeconomic circumstances do better in more equal countries.

Methods

Study design and participants

We conducted a coordinated analysis of five cohort studies from countries with different levels of income inequality to compare social gradients in height at age 4-6 years, by household socioeconomic position.

We included 3 national cohorts - the US Early Child Longitudinal Study K cohort (ECLS), the UK Millennium Cohort Study (MCS), and the Longitudinal Study of Australian Children K cohort (LSAC); and 2 regional or city cohorts - the Generation R Study (GenerationR) from Rotterdam in the Netherlands, and All Babies in Southeast Sweden (ABIS) from Southeast Sweden. At the time of data collection, the US had the highest income inequality (Gini coefficient = 37.0), followed by the UK (34.8), Australia (31.1), the Netherlands (26.8), and Sweden (22.2) (Table 1).

Three cohorts recruited pregnant women or infants, and the ECLS-K and LSAC-K recruited children at kindergarten entry. As analysis used secondary data only, no additional patient and participant involvement was conducted.

https://mc.manuscriptcentral.com/bmjpo

Confidential: For Review Only

Table 1: Cohort datasets and income inequality

	Early Child Longitudinal Study (ECLS-K)	Millennium Cohort Study (MCS)	The Longitudinal Study of Australian Children (LSAC-K)	The Generation R Study (GenerationR)	All Babies in Southeast Sweden (ABIS)
**Country (region)**	United States	United Kingdom	Australia	The Netherlands (Rotterdam)	Sweden (Southeast)
**Gini coefficient (for year of data analysed) ***	37.0 **(most unequal)**	34.8	31.1	26.8	22.2 **(most equal)**
**Year (for data analysed)**	1999	2006	2004	2008-10	2003
**First year of data collection**	1998/9	2001/2	2004	2001-2005	1997-1999
**Child age at recruitment**	4-6 years	9 months	4-5 years	During pregnancy	During pregnancy
**Child age at sweep of data analysed**	4-6 years	4-6 years	4-5 years	4-6 years	4-6 years
**Cohort sample size at age 4-6 years**	21,409	15,460	4,983	6,175	7,445

*Gini coefficients (net) from the SWIID database. The data from regional cohorts have been assigned the Gini coefficient for the whole country for this analysis. Comparable data on the Gini coefficients of regions were not available.

Sources: [25-29]

Processes

We conducted separate, coordinated analyses of each cohort using standardised methods to facilitate comparisons.

There were differences in recruitment and sample between cohorts, therefore we defined inclusion and exclusion criteria to maximise comparability of samples. Children were excluded if they were born outside the sample country/region, or if they were multiple births. Children were also excluded if they were from minority ethnic groups in the country, consistent with previous cross-national studies[13], and because average height often varies by ethnicity. The final samples were: singleton children aged 4-6 from the majority ethnic group who were born in the country/region in which the cohort took place.

Height was selected as an objective and comparable indicator of child physical health across cohorts. Parent education level and household income were selected as indicators of socioeconomic position due to their wide use in epidemiological research,[30] availability and comparability. To harmonise parent education, we defined four levels using the highest qualification of either parent. In the ECLS-K, MCS, LSAC-K, NLSCY and QLSCD these levels were: 1 – no secondary school; 2 – secondary school; 3 - post-secondary/technical; 4 – university degree or higher. In the Netherlands and Sweden parents were highly educated, so using the same categories would not capture differences in education status within these samples. We defined education categories for these cohorts which, though technically different, were chosen to have a similar meaning in terms of social status and employment opportunities as the study countries. In GenerationR and ABIS the levels were: 1 –

Confidential: For Review Only

secondary school; 2 – lower technical/vocational; 3 – higher technical/theoretical; 4 – university degree or higher.

To harmonise household income, we calculated equivalised household income in Purchasing Power Parity dollars (PPP$) at 2005 prices. We first converted household income bands to continuous values using interval regression.[31] In the ECLS-K and NLSCY we truncated the highest household incomes to improve comparability (as top income bands were lower in other cohorts). We accounted for differences in price and currency using the OECD consumer price index figures to calculate incomes at 2005 prices, then conversion to PPP$ using the OECD PPP$ exchange rates for 2005. Finally, we took account of differences in household size by equivalising income using the square root of the number of people in the household.

Statistical analysis

We analysed each dataset separately, then compared findings. As rates of missing data were generally low (0.2-2.2%), we conducted complete case analysis. All analyses were conducted using STATA 11.

Descriptive statistics were calculated for socioeconomic position, health outcomes and other socio-demographic variables. We conducted preliminary unadjusted analysis of social gradients in child height by parent education level and by quintiles of household income. We then used linear regression to take differences in age and sex into account. We regressed child height on parent education level/household income, child sex and age. We investigated the presence of different relationships by child sex using interactions between socioeconomic exposure variables and sex, and non-linear relationships with income by including a squared income term when significant. Separate models were run for each cohort and for parent education and household income (log transformed for analysis).

Finally, we predicted height from each of the models for children in the same circumstances in each cohort, using the margins command in STATA. For parent education, height was predicted for girls and boys separately, at exactly 5 years, 0 months of age, with each of the 4 levels of parent education. For household income, height was predicted for girls and boys, aged 5 years, 0 months, at the 5th, 25th, 50th, 75th and 95th percentiles of equivalised household income. These were then presented graphically to compare the slope and level of gradients between countries.

We took account of sampling and attrition in the analysis and calculation of standard errors wherever possible. In the ECLS-K, MCS and the LSAC-K, this was achieved using the svy commands in STATA. In GenerationR and ABIS, analyses were unweighted as weighting variables were not available. Associations were considered significant at the $p<0.05$ level; for decisions about whether to include second-order terms in the models (interaction and squared terms), we used a cut-off at $p<0.1$. All analyses were conducted using STATA 11.

Results

The total analytic sample was 37,063 children from five cohort studies (Table 2). The mean age ranged from 57.0 months in the LSAC-K to 72.6 months in GenerationR and the male/female proportion was similar across cohorts. Mean height varied considerably, in accordance with the age

Confidential: For Review Only

distribution across cohorts, and was highest in GenerationR. Boys were significantly taller in all cohorts. Parent education levels were highest in GenerationR and ABIS and considerably lower in the MCS; household income was considerably higher in the ECLS-K than other cohorts. Unadjusted social gradients in child height are reported in the supplementary file.

Table 2: Child and household characteristics, by cohort

	ECLS-K (US)	MCS (UK)	LSAC-K (Australia)	GenerationR (Netherlands)	ABIS (Sweden)
**Analytic sample size**	9,495	12,523	4,243	3,632	7,170
**Child age, months, mean (sd)**	68.8 (4.3)	62.6 (2.9)	57.0 (2.6)	72.6 (3.3)	64.5 (3.5)
**Sex, n girls (%)**	4,606 (48.0%)	6,097 (48.8%)	2,082 (48.7%)	1,837 (50.6%)	3,408 (47.5%)
**Height, cm, mean (sd)**	113.6 (5.4)	110.6 (4.9)	108.5 (4.7)	118.9 (5.2)	114.1 (5.2)
**Highest parent education level, n (%)**					
**Level 1 (lowest)**	132 (3.8%)	1,791 (13.7%)	280 (8.1%)	168 (4.8%)	144 (2.0%)
**Level 2**	250 (23.5%)	5,259 (42.3%)	1,042 (25.6%)	741 (21.1%)	1,344 (18.9%)
**Level 3**	633 (36.9%)	1,362 (11.1%)	1,374 (34.4%)	908 (25.8%)	2,246 (31.6%)
**Level 4 (highest)**	597 (35.9%)	3,936 (32.9%)	1,527 (31.9%)	1,701 (48.4%)	3,371 (47.5%)
**Household income, 2005 PPP$*, mean (sd)**	69,570 (48,309)	41,823 (26,163)	44,958 (22,182)	46,172 (17,092)	43,216 (16,196)

Note: n are unweighted; % are weighted in all cohorts except GenerationR and ABIS

*measured before tax in ECLS-K; measured after tax in the MCS, LSAC-K, GenerationR, ABIS. Converted to Purchasing Power Parity dollars at 2005 prices.

Parent education was a significant predictor of child height in adjusted regression analysis in all cohorts (Table 3). Gradients in child height by parent education were steepest in the cohorts from the most unequal countries (ECLS-K and MCS), with marked, but less steep gradients in the cohorts from more equal countries (LSAC-K, GenerationR, ABIS). In ABIS, the gradient was almost flat except for the lowest education category (which contained only 2% of children). Boys were significantly taller in all cohorts, and there was no significant interaction between sex and parent education. Figure 1 shows predicted gradients in child height from each model for girls only aged exactly 5 years, by parent education level. Girls are shown as there was no sex- parent education interaction, so patterns were the same for both sexes.

Equivalised household income was also a significant predictor of child height in all cohorts (Table 4). Gradients were steepest in the cohorts from the three most unequal countries (ECLS-K, MCS, LSAC-K), and less steep in the cohorts from the most equal countries (ABIS and GenerationR). There was

Confidential: For Review Only

no significant interaction between sex and household income. Figure 2 shows predicted gradients in child height from each model for girls aged exactly 5 years, by household income level.

Children were taller in the cohorts from the most equal countries at every parent education or income level, with the greatest difference between cohorts among children with the least educated parents (a 'fanning out' pattern; see Figures 1 and 2).

Confidential: For Review Only

Table 3: Multivariable regression models of child height, parent education level, child age and sex

	ECLS-K (US, Gini=37.0)	MCS (UK, Gini=34.8)	LSAC-K (Australia, Gini=31.1)	GenerationR (Netherlands, Gini=26.8)	ABIS (Sweden, Gini=22.2)
**Parent education**					
Level 1	-1.88 (-2.56 : -1.21)	-1.60 (-1.90 : -1.29)	-0.84 (-1.42 : -0.26)	-0.84 (-1.63 : -0.07)	-1.28 (-2.14 : -0.42)
Level 2	-1.00 (-1.26 : -0.74)	-0.99 (-1.24 : -0.73)	-0.46 (-0.82 : -0.11)	-0.44 (-0.87 : -0.02)	-0.12 (-0.43 : 0.20)
Level 3	-0.34 (-0.62 : -0.07)	-0.72 (-1.07 : -0.36)	-0.39 (-0.71 : -0.07)	-0.34 (-0.74 : -0.05)	-0.11 (-0.37 : 0.16)
Level 4 (baseline)	0.00	0.00	0.00	0.00	0.00
(Wald test for difference between parent education categories)	p<0.01	p<0.01	p<0.01	p=0.04	p<0.01
**Age (months)**	0.47 (0.44 : 0.50)	0.55 (0.52 : 0.58)	0.52 (0.46 : 0.57)	0.58 (0.53 : 0.63)	0.56 (0.52 : 0.59)
**Sex - boy**	0.83 (0.59 : 1.07)	0.96 (0.75 : 1.17)	1.01 (0.73 : 1.29)	0.63 (0.30 : 0.95)	0.90 (0.67 : 1.14)
**No. observations**	9,282	12,182	4,191	3,512	6,464

[Figure 1 here]

Table 4: Multivariable regression models of child height, household income, child age and sex

	ECLS-K (US, Gini=37.0)	MCS (UK, Gini=34.8)	LSAC-K (Australia, Gini=31.1)	GenerationR (Netherlands, Gini=26.8)	ABIS (Sweden, Gini=22.2)
**Log equiv. household income (PPP$)**	-0.95 (-1.56 : -0.34)	0.85 (0.69 : 1.01)	0.89 (0.63 : 1.15)	0.71 (0.29 : 1.13)	0.68 (0.35 : 1.01)
**Log equiv. household income (PPP$) ^2**	0.08 (0.04 : 0.11)				
**Age (months)**	0.47 (0.45 : 0.50)	0.55 (0.52 : 0.58)	0.52 (0.46 : 0.58)	0.58 (0.53 : 0.63)	0.56 (0.52 : 0.59)
**Sex - boy**	0.82 (0.57 : 1.06)	0.97 (0.76 : 1.18)	1.02 (0.73 : 1.31)	0.58 (0.25 : 0.91)	0.91 (0.68 -:1.14)
**No. observations**	9,257	12,170	4,073	3,311	6,455

[Figure 2 here]

Discussion

We compared social gradients in child height to explore whether children in all socioeconomic circumstances do better in more equal countries. After adjusting for differences in age and sex, children were shorter on average and the social gradient was steepest in the cohorts from the most unequal countries. A 'fanning out' pattern was evident, with children in more equal countries taller at all levels of parent education, but with the greatest between-country differences among children with less educated parents. Analysis of social gradients in relation to household income indicated broadly similar patterns.

Findings from comparison of harmonised cohort studies may reflect methodological differences between the studies, errors in the comparative method, or actual population differences.[32] Our coordinated approach was a strength, enabling a high degree of harmonisation of samples and variables, increasing confidence that findings reflect actual population differences. By including only singleton children aged 4-6 from the majority ethnic group, we minimised the extent to which comparative findings can be explained by differences in age or ethnicity of children between the cohort samples. However, as the Swedish and Dutch cohorts had subnational samples, findings may not be representative of the country as a whole. In particular, GenerationR is from a city with relatively high inequality and poverty in relation to other parts of the Netherlands.[33] Some differences in harmonised variables may also have limited comparisons. Parent education categories were chosen to have a similar social meaning, but were technically different between countries. Using identical categories would have resulted in over 95% of children being in the top two categories in ABIS and GenerationR. Finally, incomes in the MCS, LSAC-K and GenerationR were measured after tax, however incomes in ECLS-K were measured before tax (so may be higher and ordered differently than if they were measured after tax).

A relationship between income inequality and height has previously been established. In a recent analysis of data from 169 countries, income inequality was been shown to be a greater predictor of average height than absolute national income. [34] Our findings provide further evidence in support of previous studies showing better health among people at all points in the social hierarchy in more equal countries.[11-16]

To understand the meaning of differences in height between countries and by socioeconomic position, the relative contributions of genetic height potential and environmental conditions need to be unpicked. Although, at an individual level, genetic differences explain the majority of variation in body height,[35] when comparing populations (e.g. socioeconomic groups or country populations), environmental factors are thought to play the largest role.[36] Population heights have increased over time, alongside improvements in living standards within countries, and height has often been used as a marker of societal living conditions.[37 38] The Dutch population, for example, is now the tallest in the world after an increase in average height of over 20 cm since the 1850s.[39] A number of international studies have shown little variation between populations and ethnic groups in the

Confidential: For Review Only

growth of infants and children from affluent, educated families, or when nutritional and health needs are met.[40-43] This suggests that when environmental conditions are optimal, genetic factors play a limited role in ethnic variations in height. But living standards in the study countries vary, and interpreting the relative contribution of genetic potential and socioeconomic differences in this context is complex. In the UK, for example, children from Asian and Black backgrounds are taller than white children, despite living in more deprived areas.[24] We therefore analysed the sample from the majority ethnic group only (white European heritage in all cohorts) to minimise concerns about differences in genetic potential, and ensure that comparisons reflect environmental differences between socioeconomic groups and countries.

There are number of potential explanations for a relationship between income inequality and the social gradient in child health. Living in an unequal country may have psychosocial effects on children and their families. Stressors of family life related to living in an unequal country, such as increased mental illness, child maltreatment and long working hours, increased status differentiation and lower social cohesion, are all likely to affect health.[1] More equal countries also often have more generous welfare systems and more equitable public infrastructure, which may influence both population health and health inequalities.[44 45] Other contextual differences between countries, such as Gross National Income may play a role, although in work not reported here we did not find a clear relation with social gradients in the study countries. Future research using different datasets and outcomes would enable further exploration of this relationship.

In the context of growing income inequality in high-income countries, it is important to understand the implications of income inequality for population health.[46] First, we have demonstrated cross-national variation in social gap in child height. This suggests that such differences are not inevitable and could be avoided through appropriate policies and interventions. These inequalities in child height are likely to have long-term implications for health and wellbeing later in life. Second, this analysis provides evidence that children across society do better in more equal societies; although the greatest benefit is among children from less advantaged backgrounds, even children within the most advantaged families may do less well in more unequal societies. Our findings suggest a picture of multiple, overlapping and interacting socioeconomic contexts (Figure 3). Children in less advantaged socioeconomic circumstances in unequal societies may experience a double detriment – from individual socioeconomic circumstances and from living in an unequal country.

[Figure 3 here]

Confidential: For Review Only

Funding statement

This work was primarily supported by a doctoral training studentship from the UK Economic and Social Research Council. Further support was provided by grants awarded by the Canadian Institutes of Health Research (#MSH95353, #MOP123533, #00309MOP-123079).

The ECLS-K is managed by the National Center for Education Statistics, US Department of Education. For the MCS, we are grateful to the Centre for Longitudinal Studies, Institute of Education for the use of these data and to the UK Data Archive and Economic and Social Data Service for making them available. However, they bear no responsibility for the analysis or interpretation of these data. The LSAC was initiated and is funded by the Australian Government Department of Families, Housing, Community Services and Indigenous Affairs (FaHCSIA), and is undertaken in partnership with the Australian Institute of Family Studies (AIFS) and the Australian Bureau of Statistics (ABS). The Generation R Study is conducted by the Erasmus Medical Center, Rotterdam in close collaboration with the School of Law and Faculty of Social Sciences of the Erasmus University Rotterdam, the Municipal Health Service Rotterdam area, the Rotterdam Homecare Foundation and the Stichting Trombosedienst & Artsenlaboratorium Rijnmond (STAR), Rotterdam. We gratefully acknowledge the contribution of general practitioners, hospitals, midwives and pharmacies in Rotterdam. The first phase of the Generation R Study was made possible by financial support from the Erasmus Medical Center, Rotterdam, the Erasmus University Rotterdam and the Netherlands Organization for Health Research and Development (ZonMw). We are grateful to the ABIS team at Linkoping University, all families, well-baby clinics and schools. ABIS has been funded by Swedish Research Council (No. K2009-70X-21086-01-3), grants from The Swedish Council for Working Life and Social Research (No. 2008-0284), Medical Research Council of Southeast Sweden, Swedish Child Diabetes Foundation (Barndiabetesfonden), Juvenile Diabetes Research Foundation, and Research Council for Southeast Sweden.

The funders of the study had no role in the design of the study, collection, analysis, and interpretation of data, or in writing this manuscript.

For peer review only

Competing interests

TF, VWVJ, JL, HR, LS, AIW and JJM report no conflicts of interest. PKB reports funding from an ESRC doctoral studentship and the CIHR. HG reports funding from an ESRC doctoral studentship. KEP reports grants from the ESRC during the conduct of the study, and is co-founder and trustee of The Equality Trust, which campaigns for greater income equality.

Authors' contributions

PKB contributed to the study design, conducted the data analysis and literature search and drafted the report. KEP and HG contributed to the study design, interpretation of findings and writing of the report. TF, VWVJ, JL, HR, LS, AIW and JJM contributed to access to the cohort data, harmonisation of data, interpretation of findings and writing the report. All authors edited the report and approved the final draft.

Ethics approval and consent to participate

The study involved analysis of secondary data only. All cohort studies had been reviewed and approved by appropriate ethics review boards and obtained informed consent from participants.

Acknowledgements

We would like to thank Jonathan Bradshaw and members of the International Network for Research on Inequalities in Child Health (INRICH) for advice and comments on the study.

Confidential: For Review Only

References

1. Wilkinson RG, Pickett KE. Income inequality and population health: A review and explanation of the evidence. *Soc Sci Med* 2006;62(7):1768-84. doi: DOI: 10.1016/j.socscimed.2005.08.036
2. Pickett KE, Wilkinson RG. Income inequality and health: a causal review. *Soc Sci Med* 2015;128:316-26. doi: 10.1016/j.socscimed.2014.12.031 [published Online First: 2015/01/13]
3. Pickett KE, Wilkinson RG. Child wellbeing and income inequality in rich societies: ecological cross sectional study. *BMJ* 2007;335(7629):1080. doi: 10.1136/bmj.39377.580162.55
4. Wilkinson RG, Pickett KE. The Spirit Level. Why equality is better for everyone. London: Penguin Books 2009.
5. Gold R, Kennedy B, Connell F, et al. Teen births, income inequality, and social capital: developing an understanding of the causal pathway. *Health Place* 2002;8(2):77-83. doi: 10.1016/s1353-8292(01)00027-2
6. Macinko JA, Shi L, Starfield B. Wage inequality, the health system, and infant mortality in wealthy industrialized countries, 1970-1996. *Soc Sci Med* 2004;58(2):279-92. doi: Doi: 10.1016/s0277-9536(03)00200-4
7. Mayer SE, Sarin A. Some mechanisms linking economic inequality and infant mortality. *Soc Sci Med* 2005;60(3):439-55. doi: 10.1016/j.socscimed.2004.06.005
8. Pickett KE, Wilkinson RG. The Ethical and Policy Implications of Research on Income Inequality and Child Well-Being. *Pediatrics* 2015;135(Supplement 2):S39-S47. doi: 10.1542/peds.2014-3549E
9. Marmot M, Allen J, Goldblatt P, et al. Fair society, healthy lives: strategic review of health inequalities in England post 2010. London: The Marmot Review, 2010.
10. Wilkinson RG, Pickett KE. Income Inequality and Socioeconomic Gradients in Mortality. *Am J Public Health* 2008;98(4):699-704. doi: 10.2105/ajph.2007.109637
11. Leon DA, Vågerö D, Olausson PO. Social class differences in infant mortality in Sweden: comparison with England and Wales. *BMJ* 1992;305(6855):687-91. doi: 10.1136/bmj.305.6855.687
12. Vagerö D, Lundberg O. Health inequalities in Britain and Sweden. *Lancet* 1989;334(8653):35-36. doi: Doi: 10.1016/s0140-6736(89)90266-3
13. Banks J, Marmot M, Oldfield Z, et al. Disease and Disadvantage in the United States and in England. *JAMA* 2006;295(17):2037-45. doi: 10.1001/jama.295.17.2037
14. Guarnizo-Herreño CC, Watt RG, Pikhart H, et al. Socioeconomic inequalities in oral health in different European welfare state regimes. *Journal of Epidemiology and Community Health* 2013 doi: 10.1136/jech-2013-202714
15. McDonough P, Worts D, Sacker A. Socioeconomic inequalities in health dynamics: A comparison of Britain and the United States. *Social Science & Medicine* 2010;70(2):251-60. doi: http://dx.doi.org/10.1016/j.socscimed.2009.10.001
16. Siddiqi A, Brown R, Nguyen QC, et al. Cross-national comparison of socioeconomic inequalities in obesity in the United States and Canada. *International Journal for Equity in Health* 2015;14(1):1-10. doi: 10.1186/s12939-015-0251-2
17. Elani HW, Harper S, Thomson WM, et al. Social inequalities in tooth loss: A multinational comparison. *Community dentistry and oral epidemiology* 2017;45(3):266-74. doi: 10.1111/cdoe.12285 [published Online First: 2017/02/12]
18. Montgomery SM, Bartley MJ, Wilkinson RG. Family conflict and slow growth. *Archives of Disease in Childhood* 1997;77(4):326-30. doi: 10.1136/adc.77.4.326
19. Commission on Social Determinants of Health. Closing the gap in a generation: Health equity through action on the social determinants of health. Final Report of the Commission on Social Determinants of Health. Geneva: World Health Organization, 2008.

Confidential: For Review Only

20. Anne Case, Christina Paxson. Stature and Status: Height, Ability, and Labor Market Outcomes. *Journal of Political Economy* 2008;116(3):499-532. doi: doi:10.1086/589524
21. Judge TA, Cable DM. The effect of physical height on workplace success and income: preliminary test of a theoretical model: American Psychological Association, 2004.
22. Whitley E, Martin RM, Davey Smith G, et al. The association of childhood height, leg length and other measures of skeletal growth with adult cardiovascular disease: the Boyd–Orr cohort. *Journal of Epidemiology and Community Health* 2012;66(1):18-23. doi: 10.1136/jech.2009.104216
23. Howe LD, Tilling K, Galobardes B, et al. Socioeconomic differences in childhood growth trajectories: at what age do height inequalities emerge? *J Epidemiol Community Health* 2012;66(2):143-8. doi: 10.1136/jech.2010.113068 [published Online First: 2010/08/21]
24. Hancock C, Bettiol S, Smith L. Socioeconomic variation in height: analysis of National Child Measurement Programme data for England. *Arch Dis Child* 2016;101(5):422-6. doi: 10.1136/archdischild-2015-308431 [published Online First: 2015/09/06]
25. Tourangeau K, Nord C, Lê T, et al. Early Childhood Longitudinal Study, Kindergarten Class of 1998–99 (ECLS-K), Combined User's Manual for the ECLS-K Eighth-Grade and K–8 Full Sample Data Files and Electronic Codebooks (NCES 2009–004). Washington, DC: National Center for Education Statistics, Institute of Education Sciences, U.S. Department of Education, 2009.
26. Hansen K, editor. *Millennium Cohort Study. First, second, third and fourth Surveys. A guide to the datasets (seventh edition)*. London: Centre for Longitudinal Studies, 2012.
27. Australian Institute of Family Studies. Longitudinal Study of Australian Children Data User Guide - August 2011. Melbourne, 2011.
28. Jaddoe VV, van Duijn C, Franco O, et al. The Generation R Study: design and cohort update 2012. *Eur J Epidemiol* 2012;27(9):739-56. doi: 10.1007/s10654-012-9735-1
29. Ludvigsson J, Gustafsson-Stolt U, Liss P-E, et al. Mothers of Children in ABIS, a Population-Based Screening for Prediabetes, Experience Few Ethical Conflicts and Have a Positive Attitude. *Annals of the New York Academy of Sciences* 2002;958(1):376-81. doi: 10.1111/j.1749-6632.2002.tb03007.x
30. Galobardes B, Shaw M, Lawlor DA, et al. Indicators of socioeconomic position (part 1). *Journal of epidemiology and community health* 2006;60(1):7-12. doi: 10.1136/jech.2004.023531
31. Ketende SC, Joshi H. Income and poverty. In: Hansen K, Joshi H, eds. Millennium Cohort Study Third Survey: A User's Guide to Initial Findings. London Centre for Longitudinal Studies, Institute of Education, University of London 2008.
32. Bath PA, Deeg D, Poppelaars J. The harmonisation of longitudinal data: a case study using data from cohort studies in The Netherlands and the United Kingdom. *Ageing Soc* 2010;30(08):1419-37. doi: doi:10.1017/S0144686X1000070X
33. GGD Rotterdam-Rijnmond. Rotterdam: A Healthy City. Public Health Policy Memorandum Rotterdam 2011 - 2014. Rotterdam: GGD Rotterdam-Rijnmond, 2011.
34. Bogin B, Scheffler C, Hermanussen M. Global effects of income and income inequality on adult height and sexual dimorphism in height. *American Journal of Human Biology* 2017;29(2):e22980-n/a. doi: 10.1002/ajhb.22980
35. Silventoinen K. Determinants of variation in adult body height. *Journal of biosocial science* 2003;35(2):263-85. [published Online First: 2003/04/01]
36. Steckel RH. Stature and the Standard of Living. *Journal of Economic Literature* 1995;33:1903-40.
37. Blum M. Inequality and Heights. The Oxford Handbook of Economics and Human Biology: Oxford University Press 2015.
38. Garcia J, Quintana-Domeque C. The evolution of adult height in Europe: A brief note. *Economics & Human Biology* 2007;5(2):340-49. doi: https://doi.org/10.1016/j.ehb.2007.02.002
39. Schönbeck Y, Talma H, van Dommelen P, et al. The world's tallest nation has stopped growing taller: the height of Dutch children from 1955 to 2009. *Pediatric Research* 2012;73:371. doi: 10.1038/pr.2012.189

Confidential: For Review Only

40. de Onis M, Branca F. Childhood stunting: a global perspective. *Maternal & Child Nutrition* 2016;12:12-26. doi: 10.1111/mcn.12231

41. Habicht J-P, Yarbrough C, Martorell R, et al. Height and weight standards for preschool children: How relevant are ethnic differences in growth potential? . *The Lancet* 1974;303(7858):611-15. doi: https://doi.org/10.1016/S0140-6736(74)92663-4

42. Villar J, Papageorghiou AT, Pang R, et al. The likeness of fetal growth and newborn size across non-isolated populations in the INTERGROWTH-21st Project: the Fetal Growth Longitudinal Study and Newborn Cross-Sectional Study. *The lancet Diabetes & endocrinology* 2014;2(10):781-92. doi: 10.1016/s2213-8587(14)70121-4 [published Online First: 2014/07/11]

43. WHO Multicentre Growth Reference Study Group. Assessment of differences in linear growth among populations in the WHO Multicentre Growth Reference Study. *Acta paediatrica (Oslo, Norway : 1992) Supplement* 2006;450:56-65. [published Online First: 2006/07/05]

44. Eikemo TA, Bambra C, Judge K, et al. Welfare state regimes and differences in self-perceived health in Europe: A multilevel analysis. *Soc Sci Med* 2008;66(11):2281-95. doi: http://dx.doi.org/10.1016/j.socscimed.2008.01.022

45. Lynch JW, Davey Smith G, Kaplan GA, et al. Income inequality and mortality: importance to health of individual income, psychosocial environment, or material conditions. *BMJ* 2000;320(7243):1200-4. [published Online First: 2000/04/28]

46. OECD. Divided We Stand: Why Inequality Keeps Rising OECD publishing, 2011.

Confidential: For Review Only

Figure 1: Predicted gradients in child height for girls aged exactly 5 years, by parent education level

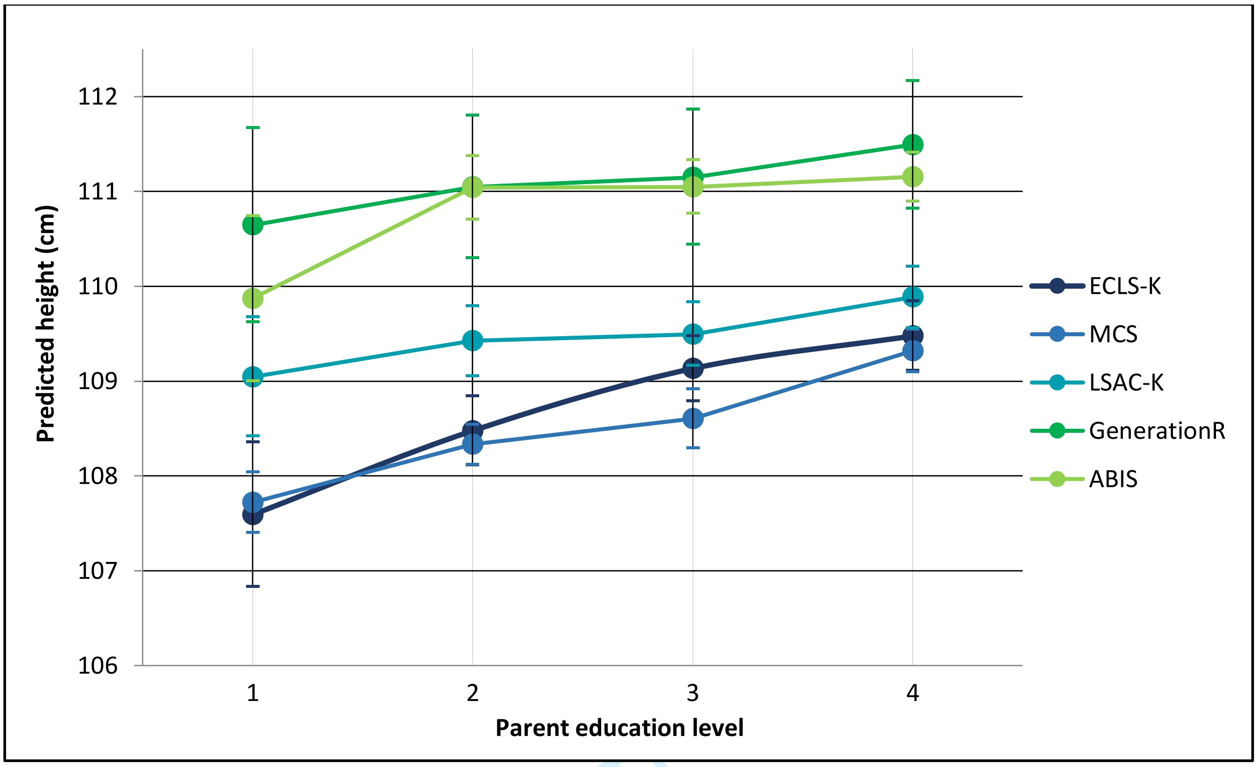

For Review Only

Figure 2: Predicted gradients in child height for girls aged exactly 5 years, by equivalised household income percentile (p5-p95)

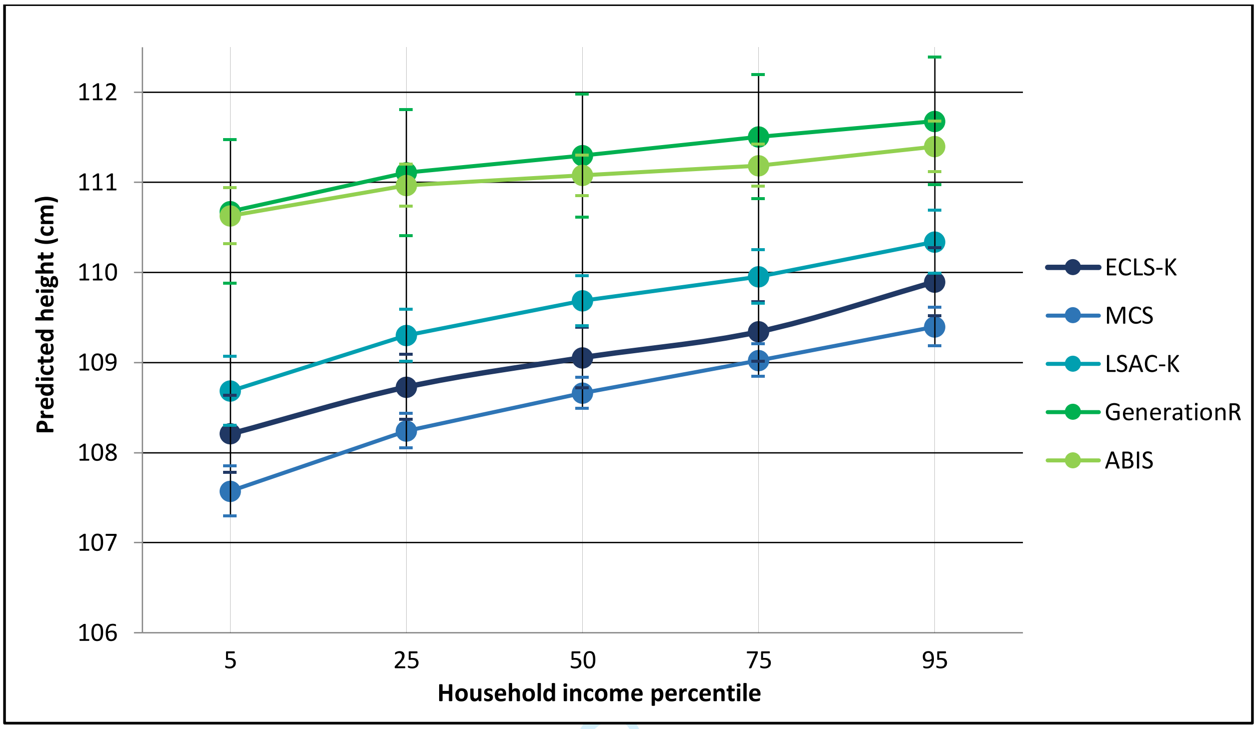

For Review Only

Figure 3: Layers of child health and development detriment

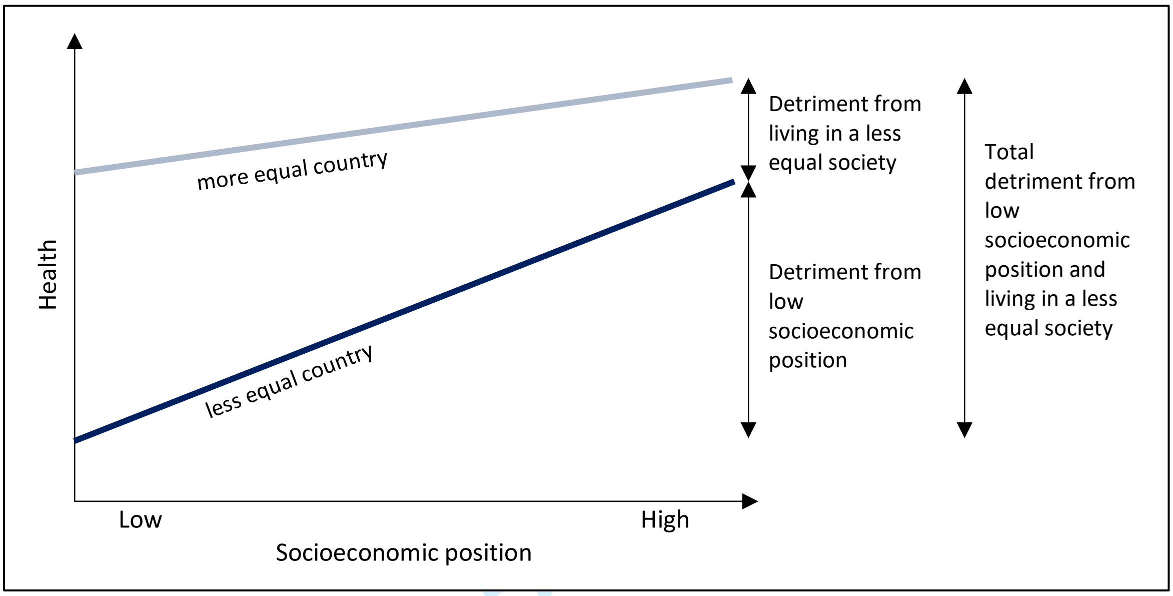

For Review Only

Supplementary file

Supplementary Table 1a: Parental education response categories in each cohort and recoding strategies

Harmonised parental education	ECLS-K (US)	MCS (UK)	LSAC-K (Australia)
Level 1 (Below secondary school qualification/ not completed school)	8th grade or below<br>9th-12th grade	Academic qualification either of: CSE below grade 1/GCSE or O Level below grade C, SCE Standard, Ordinary grades below grade 3, or Junior Certificate below grade C<br>Or vocational qualification any of: NVQ or SVQ Level 1/GNVQ Foundation Level or GSVQ Level 1, BTEC, SCOTVEC first or general certificate/SCOTVEC modules, City & Guilds part 1/RSA Stage I,II,III/Junior certificate	Years of schooling <=10 AND highest qualification = has no certificate
Level 2 (Completed secondary school qualification)	High school diploma/ equivalent	Academic qualification either of: A/AS/S Levels/SCE Higher, Scottish Certificate Sixth Year Studies, Leaving Certificate or equivalent, Level or GCSE grade A-C, SCE Standard, Ordinary grades 1-3, or Junior Certificate grade A-C<br>Or vocational qualification any of: NVQ or SVQ Level 3/GNVQ Advanced or GSVQ Level 3, OND, ONCM BTEC National, SCOTVEC National Certificate, City & Guilds advanced craft, Part III/RSA Advanced Diploma, NVQ or SVQ Level 2/GNVQ Intermediate or GSVQ Level 2, BTEC, SCOTVEC first or general diploma, City & Guilds Craft or Part II/RSA Diploma	Years of schooling >=10 AND highest qualification = has a certificate
Level 3 (Post-secondary or technical qualification)	Vocational/ technical programme<br>Some college<br>Graduate/ professional school – no degree	Academic qualification either of: Post-graduate Diplomas and Certificates, Diplomas in higher education and other higher education qualifications, Teaching qualifications for schools or further education (below degree level)<br>Or vocational qualification any of: Nursing or other medical qualifications (below degree level), NVQ or SVQ level 4 or 5, HND, HNC, Higher Level BTEC/RSA Higher Diploma	Has trade qualification or highest qualification = advanced diploma/diploma
Level 4 (Degree or higher qualification)	Bachelor's degree<br>Masters degree<br>Doctorate/professional degree	Academic qualification either of: Higher Degree and Postgraduate qualifications, First Degree (including B.Ed.)<br>Or vocational qualification any of: Professional qualifications at degree level e.g. graduate member of professional institute, chartered accountant or surveyor	Highest qualification = bachelor degree or graduate diploma/certificate or postgraduate degree

Confidential: For Review Only

Supplementary Table 1b: Parental education response categories in each cohort and recoding strategies

Harmonised parental education variable	GenerationR (Netherlands)	ABIS (Sweden)
Level 1(Completed secondary school qualification)	No primary school completed<br>Primary school completed (elementary education)<br>Special/remedial primary education (ZMOK, MLK, ZMLK, BLO, LOM)<br>Special secondary education (VSO-LOM, VSO-MLK)<br>Pre-vocational education (VBO)	Grundskola (elementary/ secondary school)
Level 2 (Lower technical/vocational qualification)	Lower vocational education / secondary general education (VMBO, MAVO, MULO)<br>General secondary education (HAVO, VWO, HBS, MMS, lyceum, atheneum, gymnasium)	Gymnasium, praktisk linje (senior high school – practical)
Level 3 (Higher technical/theoretical qualification)	Senior secondary vocational education (MBO, apprenticeship training)<br>Higher professional education (HBO	Gymnasium, teoretisk linje (senior high school - theoretical<br>Folkhögskola (residential college)
Level 4 (Degree or higher qualification)	University degree (WO)	Högskolautbildning högskole/univ-utbildning (college/university)

Supplementary Table 2a: Unadjusted gradients in child height by highest parental education level

	ECLS-K (US)		MCS (UK)		LSAC-K (Australia)		GenerationR (Netherlands)		ABIS (Sweden)	
	n	mean	n	mean	n	mean	n	mean	n	mean
**Girls**										
Level 1 (lowest)	127	111.7	876	109.1	147	107.6	74	118.2	58	112.5
Level 2	974	112.6	2498	109.9	502	107.7	371	118.4	606	113.5
Level 3	1721	113.1	660	110.0	663	108.1	456	118.4	1053	113.3
Level 4 (highest)	1697	113.3	1912	110.7	741	108.4	869	118.7	1585	113.8
Total	4519	113.0	5946	110.1	2053	108.0	1770	118.5	3302	113.6
**Boys**										
Level 1 (lowest)	159	113.4	873	110.0	129	108.6	94	119.7	77	113.5
Level 2	1042	113.6	2688	110.7	525	108.9	367	119.3	705	114.4
Level 3	1720	114.1	683	111.2	705	108.9	451	119.0	1154	114.5
Level 4 (highest)	1843	114.6	1992	111.8	779	109.3	830	119.3	1726	114.6
Total	4764	114.2	6236	111.0	2138	109.0	1742	119.2	3662	114.5

Note: Numbers are unweighted, means are weighted (unweighted in GenerationR and ABIS).

Confidential: For Review Only

Supplementary Table 2b: Unadjusted gradients in child height by equivalised household income quintile

	ECLS-K (US)		MCS (UK)		LSAC-K (Australia)		GenerationR (Netherlands)		ABIS (Sweden)	
**Household income quintile**	n	mean	n	mean	n	mean	n	mean	n	mean
**Girls**										
1 (lowest)	836	112.4	1261	109.4	358	107.3	317	118.4	625	113.0
2	883	112.9	1259	109.6	386	107.6	340	118.6	658	113.5
3	905	113.4	1181	110.1	386	108.0	327	119.0	680	113.3
4	942	113.2	1150	110.3	402	108.7	342	118.2	680	113.5
5 (highest)	953	113.2	1089	110.8	462	108.5	334	118.7	665	114.3
Total	4519	113.0	5940	110.1	1994	108.0	1660	118.6	3308	113.6
**Boys**										
1 (lowest)	845	113.4	1296	110.2	380	108.5	345	119.2	762	114.2
2	947	114.2	1324	110.6	401	108.6	322	118.9	740	114.4
3	959	114.3	1268	111.0	430	108.9	336	119.2	716	114.6
4	1000	114.3	1213	111.4	427	109.1	321	119.4	712	114.4
5 (highest)	1013	114.6	1129	111.8	441	109.8	327	119.4	724	114.9
Total	4764	114.2	6230	111.0	2079	109.0	1651	119.2	3654	114.5

Note: Numbers are unweighted, means are weighted (unweighted in GenerationR).

Confidential: For Review Only

BMJ Paediatrics Open

Income inequality and social gradients in children's height: a comparison of cohort studies from five high-income countries

Journal:	*BMJ Paediatrics Open*
Manuscript ID	bmjpo-2019-000568.R1
Article Type:	Original research
Date Submitted by the Author:	17-Oct-2019

Complete List of Authors:	Bird, Philippa; University of York, Department of Health Sciences; Leeds Teaching Hospitals NHS Trust<br>Pickett, Kate; University of York, Department of Health Sciences<br>Graham, Hilary; University of York, Department of Health Sciences<br>Faresjö, Tomas; Linkopings universitet Institutionen for medicin och halsa, Allmänmedicin<br>Jaddoe, Vincent; Erasmus Medical Center, Generation R Study Group; Erasmus Medical Center, Department of Epidemiology<br>Ludvigsson, Johnny; Div of Pediatrics, Dept of Clin Exp Medicine, Medical Faculty, Linköping University, ; Crown Princess Victoria Children´s Hospital, Region Östergötland<br>Raat, Hein; Erasmus Medical Center, Department of Public Health<br>Seguin, Louise; Universite de Montreal, Department of Social and Preventive Medicine<br>Wijtzes, Anne; Erasmus Medical Center, Department of Public Health<br>McGrath, Jennifer; Concordia University, Department of Psychology

Keywords:	Epidemiology, Growth

SCHOLARONE™
Manuscripts

Income inequality and social gradients in children's height: a comparison of cohort studies from five high-income countries

Philippa K. Bird (PhD), Department of Health Sciences, University of York, York, UK, Leeds Teaching Hospitals NHS Trust, Leeds, UK

Kate E. Pickett (PhD), Department of Health Sciences, University of York, York, UK

Hilary Graham (PhD), Department of Health Sciences, University of York, York, UK

Tomas Faresjö (PhD), Department of Medical and Health Sciences, Linköping University, Linköping, Sweden

Vincent W.V. Jaddoe (MD), Generation R Study Group, Erasmus Medical Center, Rotterdam, The Netherlands; Department of Epidemiology, Erasmus Medical Center, Rotterdam, The Netherlands; Department of Pediatrics, Erasmus Medical Center, Rotterdam, The Netherlands

Johnny Ludvigsson (PhD), Division of Pediatrics, Department of Clinical and Experimental Medicine, Linköping University, Linköping, Sweden

Hein Raat (MD), Department of Public Health, Erasmus Medical Center, Rotterdam, The Netherlands

Louise Seguin (MD), Department of Social and Preventive Medicine, Université de Montréal, Québec, Canada

Anne I. Wijtzes (PhD), Department of Public Health, Erasmus Medical Center, Rotterdam, the Netherlands

Jennifer J. McGrath (PhD), Pediatric Public Health Psychology Laboratory, Department of Psychology, Concordia University, Montreal, Quebec, Canada

Corresponding author:

Dr Philippa K. Bird

philippa.bird@nhs.net

07811 786851

37 Rhodes Street, Shipley, Bradford, BD18 3JJ

Word count: 2856

Confidential: For Review Only

Abstract

Background

Health and wellbeing are better, on average, in countries that are more equal, but less is known about how this benefit is distributed across society. Height is a widely used, objective indicator of child health and predictor of lifelong wellbeing. We compared the level and slope of social gradients in children's height in high-income countries with different levels of income inequality, in order to investigate whether children growing up in all socioeconomic circumstances are healthier in more equal countries.

Methods

We conducted a coordinated analysis of data from five cohort studies from countries selected to represent different levels of income inequality (the US, UK, Australia, the Netherlands and Sweden). We used standardised methods to compare social gradients in children's height at age 4-6 years, by parent education status and household income. We used linear regression models and predicted height for children with the same age, sex and socioeconomic circumstances in each cohort.

Results

The total analytic sample was 37,063 children aged 4-6 years. Gradients by parent education and household income varied between cohorts and outcomes. After adjusting for differences in age and sex, children in more equal countries (Sweden, the Netherlands) were taller at all levels of parent education and household income than children in less equal countries (US, UK and Australia), with the greatest between-country differences among children with less educated parents and lowest household incomes.

Conclusions

The study provides preliminary evidence that children across society do better in more equal countries, with greatest benefit among children from the most disadvantaged socioeconomic groups.

Keywords

Income inequality, socioeconomic position, child health, health inequality, cohort study

Confidential: For Review Only

Income inequality and social gradients in child height:

a coordinated analysis of cohort studies from five high-income countries

What is known about the subject

- Countries with lower income inequality have better average health and wellbeing
- There is a social gradient in child health within countries
- There is some limited and inconsistent evidence that health is worse at all points of the social gradient in more unequal countries

What this study adds

- There is considerable variation in the social gap in child height between countries, suggesting that inequalities are not inevitable
- Children are shorter at all points of the social gradient in more unequal societies, with the greatest detriment among children from the most disadvantaged backgrounds

For Review Only

Introduction

On average, countries that are more equal have better health and wellbeing.[1,2] Comparisons of population data have shown that high-income countries with less income inequality have longer life expectancy, lower teenage pregnancy rates, lower infant mortality and better child wellbeing.[3–8] Within countries, the social gradient in health is well-established: each incremental improvement in socioeconomic circumstances brings an associated health gain.[9]

It remains unclear, however, how the health benefit of living in a more equal country is distributed across society - in other words, whether or not both rich and poor do better. One previous ecological study using aggregated mortality data for rich and poor counties within US states found that mortality rates for some causes of death were lower for everyone in more equal US states, but for causes of death with no social gradient, there was no inequality effect.[10] Several studies have used individual data to compare social gradients in health in different countries.[11–17] When they are interpreted in relation to income inequality, they provide some indication that health is better among people at all points in the social hierarchy in more equal countries. For example, a comparative study showed that infant mortality rates were higher overall and the social gradient was steeper in (less equal) England and Wales than (more equal) Sweden. Infant mortality was therefore higher in all social classes in England and Wales than in Sweden, with the greatest difference among lower social class groups.[11] However, there are inconsistencies between studies.

There is also ongoing discussion on the relationship between income inequality and health. Several recent reviews have concluded that health and wellbeing are better in more equal countries.[2,18] There are some differences by study design and outcome, with findings from longitudinal studies less consistent than cross sectional studies, and some mixed findings from studies on child health.[19,20] The causal mechanisms of the relationship are not fully understood.[21] Investigating how the relationship between income inequality and health varies in relation to socioeconomic position can help to clarify the nature of this relationship.

Understanding whether people in all socioeconomic circumstances do better in more equal countries might offer important potential policy levers to address inequality. When considering *child* health, and health-related indicators of lifelong health and wellbeing that can be measured in children, the policy implications are significant. If children's trajectories of health and wellbeing are shaped by both family socioeconomic circumstances and societal levels of inequality, then promoting more equitable health and life chances is a matter of social justice.

Child height is a well-recognised marker of children's social and environmental conditions and general population health. Economic, nutritional and health constraints early in life, as well as psychological and social stressors such as family conflict, reduce children's likelihood of achieving their genetic height potential.[22] Height not only reflects past and current socioeconomic conditions, but is also a good indicator of future wellbeing and success, including better health and workplace success and higher average income.[23–26] Within countries, numerous studies have described social gradients in growth and height from birth, through childhood and into adulthood.[27,28] However, if children from affluent families are shorter in more unequal countries than equally well-off families in more equal countries, then the need for policies aimed at inequality reduction (as well as poverty alleviation) might be brought into focus.

This study aimed to answer the question: how do social gradients in child height vary in relation to income inequality in high income countries? We used child height as a marker of current and future health and wellbeing to investigate whether children growing up in all socioeconomic circumstances do better in more equal countries.

Methods

Study design and participants

We conducted a coordinated analysis of five cohort studies from countries with different levels of income inequality, using identical statistical methods and comparable variables.[29] We compared social gradients in height at age 4-6 years, by household socioeconomic position.

We included 3 national cohorts - the US Early Child Longitudinal Study K cohort (ECLS-K), the UK Millennium Cohort Study (MCS), and the Longitudinal Study of Australian Children K cohort (LSAC-K); and 2 regional or city cohorts - the Generation R Study (GenerationR) from Rotterdam in the Netherlands, and All Babies in Southeast Sweden (ABIS) from Southeast Sweden. At the time of data collection, the US had the highest income inequality (Gini coefficient = 37.0), followed by the UK (34.8), Australia (31.1), the Netherlands (26.8), and Sweden (22.2) (Table 1).

Confidential: For Review Only

The cohorts recruited pregnant women or infants, except the US and Australian cohorts, which recruited children at kindergarten entry. As analysis used secondary data only, no additional patient and participant involvement was conducted.

Confidential: For Review Only

Table 1: Cohort datasets and income inequality

	Early Child Longitudinal Study (ECLS-K)	Millennium Cohort Study (MCS)	The Longitudinal Study of Australian Children (LSAC-K)	The Generation R Study (GenerationR)	All Babies in Southeast Sweden (ABIS)
**Country (region)**	United States	United Kingdom	Australia	The Netherlands (Rotterdam)	Sweden (Southeast)
**Gini coefficient (for year of data analysed) ***	37.0<br>**(most unequal)**	34.8	31.1	26.8	22.2<br>**(most equal)**
**Year (for data analysed)**	1999	2006	2004	2008-10	2003
**First year of data collection**	1998/9	2001/2	2004	2001-2005	1997-1999
**Child age at recruitment**	4-6 years	9 months	4-5 years	During pregnancy	During pregnancy
**Child age at sweep of data analysed**	4-6 years	4-6 years	4-5 years	4-6 years	4-6 years
**Cohort sample size at age 4-6 years**	21,409	15,460	4,983	6,175	7,445

*Gini coefficients (net) from the SWIID database. The data from regional cohorts have been assigned the Gini coefficient for the whole country for this analysis. Comparable data on the Gini coefficients of regions were not available.
Sources: [30–34]

Processes

There were differences in recruitment and sample between cohorts, therefore we defined inclusion and exclusion criteria to maximise comparability of samples. Children were excluded if they were born outside the sample country/region, or if they were multiple births. Children were also excluded if they were from minority ethnic groups in the country, consistent with previous cross-national studies [13], and because average height often varies by ethnicity. The final samples were: singleton children aged 4-6 from the majority ethnic group who were born in the country/region in which the cohort took place.

Height was selected as an objective and comparable indicator of child physical health across cohorts. Parent education level and household income were selected as indicators of socioeconomic position due to their wide use in epidemiological research,[35] availability and comparability. To harmonise

parent education, we defined four levels using the highest qualification of either parent. In the US, UK and Australian cohorts these levels were: 1 – no secondary school; 2 – secondary school; 3 - post-secondary/technical; 4 – university degree or higher. In the Netherlands and Sweden parents were highly educated, so using the same categories would not capture differences in education status within these samples. We defined education categories for these cohorts which, though technically different, were chosen to have a similar meaning in terms of social status and employment opportunities as the study countries. In the Dutch and Swedish cohorts the levels were: 1 – secondary school; 2 – lower technical/vocational; 3 – higher technical/theoretical; 4 – university degree or higher (full explanations of categories are provided in the supplementary file).

To harmonise household income, we calculated equivalised household income in Purchasing Power Parity dollars (PPP$) at 2005 prices. We first converted household income bands to continuous values using interval regression (used to model outcomes in ordered categories where the exact value of the observation is unknown).[36] In the US cohort we truncated the highest household incomes to improve comparability (as top income bands were lower in other cohorts). We accounted for differences in price and currency using the OECD consumer price index figures to calculate incomes at 2005 prices, then conversion to PPP$ using the OECD PPP$ exchange rates for 2005. Finally, we took account of differences in household size by equivalising income using the square root of the number of people in the household.

Statistical analysis

We analysed each dataset separately, then compared findings. As rates of missing data were generally low (0.2-2.2%), we conducted complete case analysis. All analyses were conducted using STATA 11.

Descriptive statistics were calculated for socioeconomic position, health outcomes and other socio-demographic variables. We conducted preliminary unadjusted analysis of child height by parent education level and by quintiles of household income for comparability between cohorts. We then used linear regression to take differences in age and sex into account. We regressed child height on parent education level/household income, child sex and age. We investigated the presence of different relationships by child sex using interactions between socioeconomic exposure variables and sex, and non-linear relationships with income by including a squared income term when significant. Separate models were run for each cohort and for parent education and household income (log transformed for analysis).

Finally, we predicted height from each of the models for children in the same circumstances in each cohort, using the margins command in STATA. For parent education, height was predicted for girls and boys separately, at exactly 5 years of age, with each of the 4 levels of parent education. For household income, height was predicted for girls and boys, aged exactly 5 years, at the 5th, 25th, 50th, 75th and 95th percentiles of equivalised household income. These were then presented graphically to compare the slope and level of gradients between countries.

We took account of sampling and attrition in the analysis and calculation of standard errors wherever possible. In the US, UK and Australian cohorts, this was achieved using the svy commands in STATA. In the Dutch and Swedish cohorts, analyses were unweighted as weighting variables were not available. Associations were considered significant at $p<0.05$; for inclusion of second-order terms in the models (interaction and squared terms), we used a cut-off at $p<0.1$.

https://mc.manuscriptcentral.com/bmjpo

Results

The total analytic sample was 37,063 children from five cohort studies (Table 2). The mean age ranged from 57.0 months in the Australian sample to 72.6 months in the Dutch sample and the male/female proportion was similar across cohorts. Mean height varied considerably, in accordance with the age distribution across cohorts, and was highest in the Dutch cohort. Boys were significantly taller in all cohorts. Parent education levels were highest in the Dutch and Swedish cohorts and considerably lower in the UK cohort; household income was considerably higher in the US cohort than other cohorts. Unadjusted social gradients in child height are reported in the supplementary file.

Confidential: For Review Only

Table 2: Child and household characteristics, by cohort

	ECLS-K (US)	MCS (UK)	LSAC-K (Australia)	GenerationR (Netherlands)	ABIS (Sweden)
**Analytic sample size**	9,495	12,523	4,243	3,632	7,170
**Child age, months, mean (sd)**	68.8 (4.3)	62.6 (2.9)	57.0 (2.6)	72.6 (3.3)	64.5 (3.5)
**Sex, n girls (%)**	4,606 (48.0%)	6,097 (48.8%)	2,082 (48.7%)	1,837 (50.6%)	3,408 (47.5%)
**Height, cm, mean (sd)**	113.6 (5.4)	110.6 (4.9)	108.5 (4.7)	118.9 (5.2)	114.1 (5.2)
**Highest parent education level, n (%)**					
**Level 1 (lowest)**	132 (3.8%)	1,791 (13.7%)	280 (8.1%)	168 (4.8%)	144 (2.0%)
**Level 2**	250 (23.5%)	5,259 (42.3%)	1,042 (25.6%)	741 (21.1%)	1,344 (18.9%)
**Level 3**	633 (36.9%)	1,362 (11.1%)	1,374 (34.4%)	908 (25.8%)	2,246 (31.6%)
**Level 4 (highest)**	597 (35.9%)	3,936 (32.9%)	1,527 (31.9%)	1,701 (48.4%)	3,371 (47.5%)
**Household income, 2005 PPP$***					
**Mean (sd)**	69,570 (48,309)	41,823 (26,163)	44,958 (22,182)	46,172 (17,092)	43,216 (16,196)
**Median**	58,620	36,428	43,540	44,281	41,954

Note: n are unweighted; % are weighted in all cohorts except GenerationR and ABIS

*measured before tax in ECLS-K; measured after tax in the MCS, LSAC-K, GenerationR, ABIS. Converted to Purchasing Power Parity dollars at 2005 prices.

Parent education was a significant predictor of child height in adjusted regression analysis in all cohorts (Table 3). Gradients in child height by parent education were steepest in the cohorts from the most unequal countries (US and UK), with marked, but less steep gradients in the cohorts from Australia and the more equal countries (the Netherlands, Sweden). In the Swedish cohort, the gradient was almost flat except for the lowest education category (which contained only 2% of children). Boys were significantly taller in all cohorts, and there was no significant interaction between sex and parent education. Figure 1 shows predicted gradients in child height from each model for girls and boys aged exactly 5 years, by parent education level.

Equivalised household income was also a significant predictor of child height in all cohorts (Table 4). Gradients were steepest in the cohorts from the three most unequal countries (US, UK, Australia), and less steep in the cohorts from the most equal countries (Sweden and the Netherlands). There was no significant interaction between sex and household income. Figure 2 shows predicted gradients in child height from each model for girls and boys aged exactly 5 years, by household income level.

Children were taller in the cohorts from the most equal countries at every parent education or income level, with the greatest difference between cohorts among children with the least educated parents (a 'fanning out' pattern; see Figures 1 and 2).

Table 3: Multivariable regression models of child height, parent education level, child age and sex

	**ECLS-K (US, Gini=37.0)**	**MCS (UK, Gini=34.8)**	**LSAC-K (Australia, Gini=31.1)**	**GenerationR (Netherlands, Gini=26.8)**	**ABIS (Sweden, Gini=22.2)**
**Parent education**					
Level 1	-1.88 (-2.56 : -1.21)	-1.60 (-1.90 : -1.29)	-0.84 (-1.42 : -0.26)	-0.84 (-1.63 : -0.07)	-1.28 (-2.14 : -0.42)
Level 2	-1.00 (-1.26 : -0.74)	-0.99 (-1.24 : -0.73)	-0.46 (-0.82 : -0.11)	-0.44 (-0.87 : -0.02)	-0.12 (-0.43 : 0.20)
Level 3	-0.34 (-0.62 : -0.07)	-0.72 (-1.07 : -0.36)	-0.39 (-0.71 : -0.07)	-0.34 (-0.74 : -0.05)	-0.11 (-0.37 : 0.16)
Level 4 (baseline)	0.00	0.00	0.00	0.00	0.00
(Wald test for difference between parent education categories)	p<0.01	p<0.01	p<0.01	p=0.04	p<0.01
**Age (months)**	0.47 (0.44 : 0.50)	0.55 (0.52 : 0.58)	0.52 (0.46 : 0.57)	0.58 (0.53 : 0.63)	0.56 (0.52 : 0.59)
**Sex - boy**	0.83 (0.59 : 1.07)	0.96 (0.75 : 1.17)	1.01 (0.73 : 1.29)	0.63 (0.30 : 0.95)	0.90 (0.67 : 1.14)
**No. observations**	9,282	12,182	4,191	3,512	6,464

[Figure 1 here]

Confidential: For Review Only

Table 4: Multivariable regression models of child height, household income, child age and sex

	ECLS-K (US, Gini=37.0)	MCS (UK, Gini=34.8)	LSAC-K (Australia, Gini=31.1)	GenerationR (Netherlands, Gini=26.8)	ABIS (Sweden, Gini=22.2)
**Log equiv. household income (PPP$)**	-0.95 (-1.56 : -0.34)	0.85 (0.69 : 1.01)	0.89 (0.63 : 1.15)	0.71 (0.29 : 1.13)	0.68 (0.35 : 1.01)
**Log equiv. household income (PPP$) ^2**	0.08 (0.04 : 0.11)				
**Age (months)**	0.47 (0.45 : 0.50)	0.55 (0.52 : 0.58)	0.52 (0.46 : 0.58)	0.58 (0.53 : 0.63)	0.56 (0.52 : 0.59)
**Sex - boy**	0.82 (0.57 : 1.06)	0.97 (0.76 : 1.18)	1.02 (0.73 : 1.31)	0.58 (0.25 : 0.91)	0.91 (0.68 -:1.14)
**No. observations**	9,257	12,170	4,073	3,311	6,455

[Figure 2 here]

Discussion

We compared social gradients in child height to explore whether children in all socioeconomic circumstances do better in more equal countries. After adjusting for differences in age and sex, children were shorter on average and the social gradient was steepest in the cohorts from the most unequal countries. A 'fanning out' pattern was evident, with children in more equal countries taller at all levels of parent education, but with the greatest between-country differences among children with less educated parents. Analysis of social gradients in relation to household income indicated broadly similar patterns.

Findings from comparison of harmonised cohort studies may reflect methodological differences between the studies, errors in the comparative method, or actual population differences.[37] We needed to analyse each cohort separately, as data access requirements did not enable pooling of the datasets. The coordinated analysis enabled a high degree of harmonisation, increasing confidence that findings reflect actual population differences. By including only singleton children aged 4-6 from the majority ethnic group, we minimised the extent to which comparative findings can be explained by differences in age or ethnicity of children between the cohort samples. However, as the Swedish and Dutch cohorts had subnational samples, findings may not be representative of the country as a whole. In particular, GenerationR is from a city with relatively high inequality and poverty in relation to other parts of the Netherlands.[38] Some differences in harmonised variables may also have limited comparisons. Parent education categories were chosen to have a similar social meaning, but were technically different between countries. Using identical categories would have resulted in over 95% of children being in the top two categories in the Swedish and Dutch cohorts. Finally, incomes in

Confidential: For Review Only

the UK, Australian, Swedish and Dutch cohorts were measured after tax, however incomes in the Australian cohort were measured before tax (so may be higher and ordered differently than if they were measured after tax).

A relationship between income inequality and height has previously been established. In a recent analysis of data from 169 countries, income inequality was been shown to be a greater predictor of average height than absolute national income.[39] Our findings also provide further evidence in support of previous studies showing better health among people at all points in the social hierarchy in more equal countries.[11–16]

To understand the meaning of differences in height between countries and by socioeconomic position, the relative contributions of genetic height potential and environmental conditions need to be unpicked. Although, at an individual level, genetic differences explain the majority of variation in body height,[40] when comparing populations (e.g. socioeconomic groups or country populations), environmental factors are thought to play the largest role.[41] Population heights have increased over time, alongside improvements in living standards within countries, and height has often been used as a marker of societal living conditions.[42,43] The Dutch population, for example, is now the tallest in the world after an increase in average height of over 20 cm since the 1850s.[44] A number of international studies have shown little variation between populations and ethnic groups in the growth of infants and children from affluent, educated families, or when nutritional and health needs are met.[45–48] This suggests that when environmental conditions are optimal, genetic factors play a limited role in ethnic variations in height.

In this study, living standards in the cohort countries vary, and interpreting the relative contribution of genetic potential and socioeconomic differences is complex. Parental height, an important predictor of child height [49], reflects this complex interplay between genetic and environmental factors. Whilst including it in the analysis would have adjusted for genetic differences, it also would have adjusted for environmental differences that our research sought to identify. There are also complex patterns and inequalities in relation to ethnicity. In the UK, for example, children from Asian and Black backgrounds are taller than white children, despite living in more deprived areas.[28] We therefore analysed the sample from the majority ethnic group only (white European heritage in all cohorts) to minimise concerns about differences in genetic potential, and ensure that comparisons reflect environmental differences between socioeconomic groups and countries. There were considerable differences between the cohorts in the country of origin and socioeconomic position of families from ethnic minority backgrounds, as well as health outcomes. Therefore, this approach enabled a clearer focus on the research question, but also resulted in patterns in some population groups not being compared, and may have affected the patterns of inequalities observed. Further research would be useful to identify and compare the extent of inequalities by ethnicity between cohorts from different countries.

There are number of potential explanations for a relationship between income inequality and the social gradient in child health. Living in an unequal country may have psychosocial effects on children and their families. Stressors of family life related to living in an unequal country, such as increased mental illness, child maltreatment and long working hours, increased status differentiation and lower social cohesion, are all likely to affect health.[1] More equal countries also often have more generous welfare systems and more equitable public infrastructure, which may influence both population health and health inequalities.[50,51] Other contextual differences between countries,

Confidential: For Review Only

such as Gross National Income may play a role, although in work not reported here we did not find a clear relation with social gradients in the study countries. Future research using different datasets and outcomes would enable further exploration of this relationship.

In the context of growing income inequality in high-income countries, it is important to understand the implications of income inequality for population health.[52] First, we have demonstrated cross-national variation in the social gap in child height. This suggests that such differences are not inevitable and could be avoided through appropriate policies and interventions. These inequalities in child height are likely to have long-term implications for health and wellbeing later in life. Second, this analysis provides evidence that children across society do better in more equal societies; although the greatest benefit is among children from less advantaged backgrounds, even children within the most advantaged families may do better in more equal societies. Our findings suggest a picture of multiple, overlapping and interacting socioeconomic contexts (Figure 3). Children in less advantaged socioeconomic circumstances in unequal societies may experience a double detriment – from individual socioeconomic circumstances and from living in an unequal country.

[Figure 3 here]

Confidential: For Re

Funding statement

This work was primarily supported by a doctoral training studentship from the UK Economic and Social Research Council. Further support was provided by grants awarded by the Canadian Institutes of Health Research (#MSH95353, #MOP123533, #00309MOP-123079).

The ECLS-K is managed by the National Center for Education Statistics, US Department of Education. For the MCS, we are grateful to the Centre for Longitudinal Studies, Institute of Education for the use of these data and to the UK Data Archive and Economic and Social Data Service for making them available. However, they bear no responsibility for the analysis or interpretation of these data. The LSAC was initiated and is funded by the Australian Government Department of Families, Housing, Community Services and Indigenous Affairs (FaHCSIA), and is undertaken in partnership with the Australian Institute of Family Studies (AIFS) and the Australian Bureau of Statistics (ABS). The Generation R Study is conducted by the Erasmus Medical Center, Rotterdam in close collaboration with the School of Law and Faculty of Social Sciences of the Erasmus University Rotterdam, the Municipal Health Service Rotterdam area, the Rotterdam Homecare Foundation and the Stichting Trombosedienst & Artsenlaboratorium Rijnmond (STAR), Rotterdam. We gratefully acknowledge the contribution of general practitioners, hospitals, midwives and pharmacies in Rotterdam. The first phase of the Generation R Study was made possible by financial support from the Erasmus Medical Center, Rotterdam, the Erasmus University Rotterdam and the Netherlands Organization for Health Research and Development (ZonMw). We are grateful to the ABIS team at Linkoping University, all families, well-baby clinics and schools. ABIS has been funded by Swedish Research Council (No. K2009-70X-21086-01-3), grants from The Swedish Council for Working Life and Social Research (No. 2008-0284), Medical Research Council of Southeast Sweden, Swedish Child Diabetes Foundation

(Barndiabetesfonden), Juvenile Diabetes Research Foundation, and Research Council for Southeast Sweden.

The funders of the study had no role in the design of the study, collection, analysis, and interpretation of data, or in writing this manuscript.

Competing interests

TF, VWVJ, JL, HR, LS, AIW and JJM report no conflicts of interest. PKB reports funding from an ESRC doctoral studentship and the CIHR. HG reports funding from an ESRC doctoral studentship. KEP reports grants from the ESRC during the conduct of the study, and is co-founder and trustee of The Equality Trust, which campaigns for greater income equality.

Authors' contributions

PKB contributed to the study design, conducted the data analysis and literature search and drafted the report. KEP and HG contributed to the study design, interpretation of findings and writing of the report. TF, VWVJ, JL, HR, LS, AIW and JJM contributed to access to the cohort data, harmonisation of data, interpretation of findings and writing the report. All authors edited the report and approved the final draft.

Ethics approval and consent to participate

The study involved analysis of secondary data only. All cohort studies had been reviewed and approved by appropriate ethics review boards and obtained informed consent from participants.

Acknowledgements

We would like to thank Jonathan Bradshaw and members of the International Network for Research on Inequalities in Child Health (INRICH) for advice and comments on the study.

Confidential: For Review Only

References

1 Wilkinson RG, Pickett KE. Income inequality and population health: A review and explanation of the evidence. *Soc Sci Med* 2006;**62**:1768–84. doi:10.1016/j.socscimed.2005.08.036

2 Pickett KE, Wilkinson RG. Income inequality and health: A causal review. *Soc Sci Med* 2015;**128**:316–26. doi:10.1016/j.socscimed.2014.12.031

3 Pickett KE, Wilkinson RG. Child wellbeing and income inequality in rich societies: ecological cross sectional study. *BMJ* 2007;**335**:1080. doi:10.1136/bmj.39377.580162.55

4 Wilkinson R, Pickett K. *The Spirit Level. Why equality is better for everyone.* London: Penguin Books 2009.

5 Gold R, Kennedy B, Connell F, *et al.* Teen births, income inequality, and social capital: developing an understanding of the causal pathway. *Health Place* 2002;**8**:77–83.http://www.ncbi.nlm.nih.gov/pubmed/11943580 (accessed 8 Oct 2019).

6 Macinko JA, Shi L, Starfield B. Wage inequality, the health system, and infant mortality in wealthy industrialized countries, 1970-1996. *Soc Sci Med* 2004;**58**:279–92. doi:10.1016/s0277-9536(03)00200-4

7 Mayer SE, Sarin A. Some mechanisms linking economic inequality and infant mortality. *Soc Sci Med* 2005;**60**:439–55. doi:10.1016/j.socscimed.2004.06.005

8 Pickett KE, Wilkinson RG. The ethical and policy implications of research on income inequality and child well-being. *Pediatrics* 2015;**135 Suppl 2**:S39-47. doi:10.1542/peds.2014-3549E

9 Marmot M, Allen J, Goldblatt P, *et al.* Fair society, healthy lives: strategic review of health inequalities in England post 2010. London: The Marmot Review. 2010.

10 Wilkinson RG, Pickett KE. Income Inequality and Socioeconomic Gradients in Mortality. *Am J Public Health* 2008;**98**:699–704. doi:10.2105/AJPH.2007.109637

11 Leon DA, Vagero D, Olausson PO. Social class differences in infant mortality in Sweden: comparison with England and Wales. *BMJ* 1992;**305**:687–91. doi:10.1136/bmj.305.6855.687

12 Vågerö D, Lundberg O. Health inequalities in Britain and Sweden. *Lancet (London, England)* 1989;**2**:35–6. doi:10.1016/s0140-6736(89)90266-3

13 Banks J, Marmot M, Oldfield Z, *et al.* Disease and Disadvantage in the United States and in England. *JAMA* 2006;**295**:2037. doi:10.1001/jama.295.17.2037

14 Guarnizo-Herreño CC, Watt RG, Pikhart H, *et al.* Socioeconomic inequalities in oral health in different European welfare state regimes. *J Epidemiol Community Health* 2013;**67**:728–35. doi:10.1136/jech-2013-202714

15 McDonough P, Worts D, Sacker A. Socioeconomic inequalities in health dynamics: A comparison of Britain and the United States. *Soc Sci Med* 2010;**70**:251–60. doi:10.1016/j.socscimed.2009.10.001

16 Siddiqi A, Brown R, Nguyen QC, *et al.* Cross-national comparison of socioeconomic inequalities in obesity in the United States and Canada. *Int J Equity Health* 2015;**14**:116. doi:10.1186/s12939-015-0251-2

17 Elani HW, Harper S, Thomson WM, *et al.* Social inequalities in tooth loss: A multinational

Confidential: For Review Only

comparison. *Community Dent Oral Epidemiol* 2017;**45**:266–74. doi:10.1111/cdoe.12285

18 Kondo N, Sembajwe G, Kawachi I, *et al.* Income inequality, mortality, and self rated health: meta-analysis of multilevel studies. *BMJ* 2009;**339**:b4471. doi:10.1136/bmj.b4471

19 Avendano M. Correlation or causation? Income inequality and infant mortality in fixed effects models in the period 1960–2008 in 34 OECD countries. *Soc Sci Med* 2012;**75**:754–60. doi:10.1016/j.socscimed.2012.04.017

20 Leigh A, Jencks C. Inequality and mortality: Long-run evidence from a panel of countries. *J Health Econ* 2007;**26**:1–24. doi:10.1016/j.jhealeco.2006.07.003

21 Präg P, Mills M, Wittek R. Income and Income Inequality as Social Determinants of Health: Do Social Comparisons Play a Role? *Eur Sociol Rev* 2014;**30**:218–29. doi:10.1093/esr/jct035

22 Montgomery SM, Bartley MJ, Wilkinson RG. Family conflict and slow growth. *Arch Dis Child* 1997;**77**:326–30. doi:10.1136/adc.77.4.326

23 Commission on Social Determinants of Health. Closing the gap in a generation: Health equity through action on the social determinants of health. Final Report of the Commission on Social Determinants of Health. Geneva: 2008.

24 Case A, Paxson C. Stature and status: Height, ability, and labor market outcomes. *J Polit Econ* 2008;**116**:499–532. doi:10.1086/589524

25 Judge TA, Cable DM. The Effect of Physical Height on Workplace Success and Income: Preliminary Test of a Theoretical Model. *J Appl Psychol* 2004;**89**:428–41. doi:10.1037/0021-9010.89.3.428

26 Whitley E, Martin RM, Davey Smith G, *et al.* The association of childhood height, leg length and other measures of skeletal growth with adult cardiovascular disease: the Boyd–Orr cohort. *J Epidemiol Community Health* 2012;**66**:18–23. doi:10.1136/jech.2009.104216

27 Howe LD, Tilling K, Galobardes B, *et al.* Socioeconomic differences in childhood growth trajectories: at what age do height inequalities emerge? *J Epidemiol Community Health* 2012;**66**:143–8. doi:10.1136/jech.2010.113068

28 Hancock C, Bettiol S, Smith L. Socioeconomic variation in height: analysis of National Child Measurement Programme data for England. *Arch Dis Child* 2016;**101**:422–6. doi:10.1136/archdischild-2015-308431

29 Hofer SM, Piccinin AM. Integrative data analysis through coordination of measurement and analysis protocol across independent longitudinal studies. *Psychol Methods* 2009;**14**:150–64. doi:10.1037/a0015566

30 Tourangeau K, Nord C, Lê T, *et al.* Early Childhood Longitudinal Study, Kindergarten Class of 1998–99 (ECLS-K), Combined User's Manual for the ECLS-K Eighth-Grade and K–8 Full Sample Data Files and Electronic Codebooks (NCES 2009–004). Washington DC: 2009.

31 Hansen K (editor). Millennium Cohort Study. First, second, third and fourth Surveys. A guide to the datasets (seventh edition). . London: 2012.

32 Australian Institute of Family Studies. Longitudinal Study of Australian Children Data User Guide - August 2011. Melbourne: 2011.

33 Jaddoe VW V., van Duijn CM, Franco OH, *et al.* The Generation R Study: design and cohort update 2012. *Eur J Epidemiol* 2012;**27**:739–56. doi:10.1007/s10654-012-9735-1

Confidential: For Review Only

34 Ludvigsson J, Gustafsson-Stolt U, Liss P-E, *et al.* Mothers of Children in ABIS, a Population-Based Screening for Prediabetes, Experience Few Ethical Conflicts and Have a Positive Attitude. *Ann N Y Acad Sci* 2006;**958**:376–81. doi:10.1111/j.1749-6632.2002.tb03007.x

35 Galobardes B, Shaw M, Lawlor DA, *et al.* Indicators of socioeconomic position (part 1). *J Epidemiol Community Heal* 2006;**60**:7–12. doi:10.1136/jech.2004.023531

36 Ketende S, Joshi H. Income and poverty. In: Hansen K, Joshi H, eds. *Millennium Cohort Study Third Survey: A User's Guide to Initial Findings. London Centre for Longitudinal Studies*. London: : Institute of Education, University of London 2008.

37 Bath PA, Deeg D, Poppelaars J. The harmonisation of longitudinal data: a case study using data from cohort studies in The Netherlands and the United Kingdom. *Ageing Soc* 2010;**30**:1419–37. doi:10.1017/S0144686X1000070X

38 GGD Rotterdam-Rijnmond. Rotterdam: A Healthy City. Public Health Policy Memorandum Rotterdam 2011 - 2014. Rotterdam: 2011.

39 Bogin B, Scheffler C, Hermanussen M. Global effects of income and income inequality on adult height and sexual dimorphism in height. *Am J Hum Biol* 2017;**29**:e22980. doi:10.1002/ajhb.22980

40 Silventoinen K. Determinants of variation in adult body height. *J Biosoc Sci* 2003;**35**:263–85. doi:10.1017/s0021932003002633

41 Steckel RH. Stature and the Standard of Living. *J Econ Lit*;**33**:1903–40. doi:10.2307/2729317

42 Blum M. *Inequality and Heights. The Oxford Handbook of Economics and Human Biology:* Oxford: : Oxford University Press 2015.

43 Garcia J, Quintana-Domeque C. The evolution of adult height in Europe: A brief note. *Econ Hum Biol* 2007;**5**:340–9. doi:10.1016/J.EHB.2007.02.002

44 Schönbeck Y, Talma H, van Dommelen P, *et al.* The world's tallest nation has stopped growing taller: the height of Dutch children from 1955 to 2009. *Pediatr Res* 2013;**73**:371–7. doi:10.1038/pr.2012.189

45 de Onis M, Branca F. Childhood stunting: a global perspective. *Matern Child Nutr* 2016;**12**:12–26. doi:10.1111/mcn.12231

46 Habicht JP, Martorell R, Yarbrough C, *et al.* Height and weight standards for preschool children. How relevant are ethnic differences in growth potential? *Lancet (London, England)* 1974;**1**:611–4. doi:10.1016/s0140-6736(74)92663-4

47 Villar J, Papageorghiou AT, Pang R, *et al.* The likeness of fetal growth and newborn size across non-isolated populations in the INTERGROWTH-21st Project: the Fetal Growth Longitudinal Study and Newborn Cross-Sectional Study. *Lancet Diabetes Endocrinol* 2014;**2**:781–92. doi:10.1016/S2213-8587(14)70121-4

48 WHO Multicentre Growth Reference Study Group. Assessment of differences in linear growth among populations in the WHO Multicentre Growth Reference Study. *Acta Paediatr Suppl* 2006;**450**:56–65.http://www.ncbi.nlm.nih.gov/pubmed/16817679 (accessed 8 Oct 2019).

49 Galobardes B, McCormack VA, McCarron P, *et al.* Social inequalities in height: persisting differences today depend upon height of the parents. *PLoS One* 2012;**7**:e29118. doi:10.1371/journal.pone.0029118

50 Eikemo TA, Bambra C, Judge K, *et al.* Welfare state regimes and differences in self-perceived

Confidential: For Review Only

health in Europe: A multilevel analysis. *Soc Sci Med* 2008;**66**:2281–95. doi:10.1016/j.socscimed.2008.01.022

51 Lynch JW, Smith GD, Kaplan GA, *et al.* Income inequality and mortality: importance to health of individual income, psychosocial environment, or material conditions. *BMJ* 2000;**320**:1200–4. doi:10.1136/bmj.320.7243.1200

52 OECD. Divided We Stand: Why Inequality Keeps Rising. 2011.

Confidential: For Review Only

Figure 1: Predicted gradients in child height for a) girls, b) boys aged exactly 5 years, by parent education level

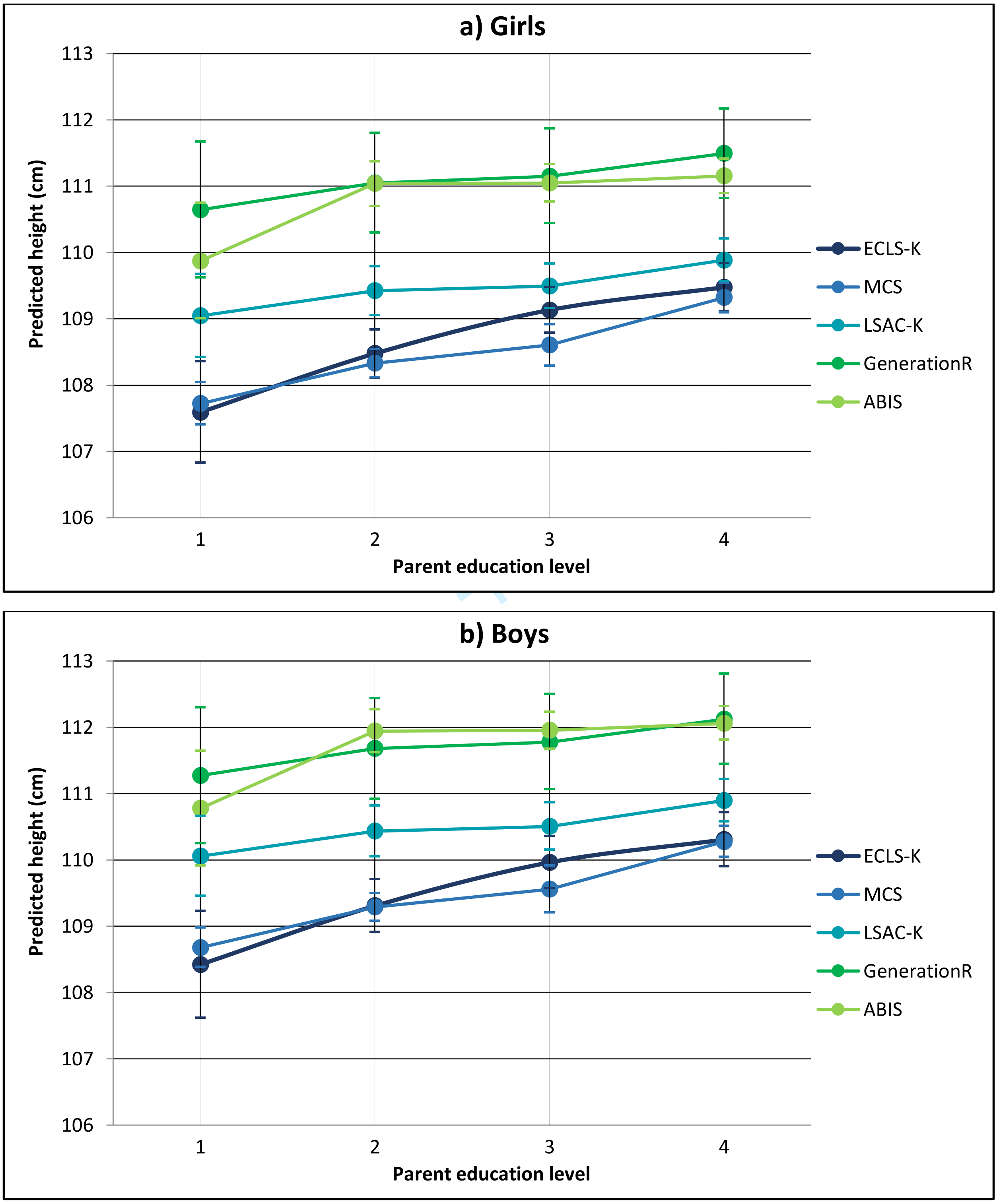

Figure 2: Predicted gradients in child height for a) girls, b) boys aged exactly 5 years, by equivalised household income percentile (p5-p95)

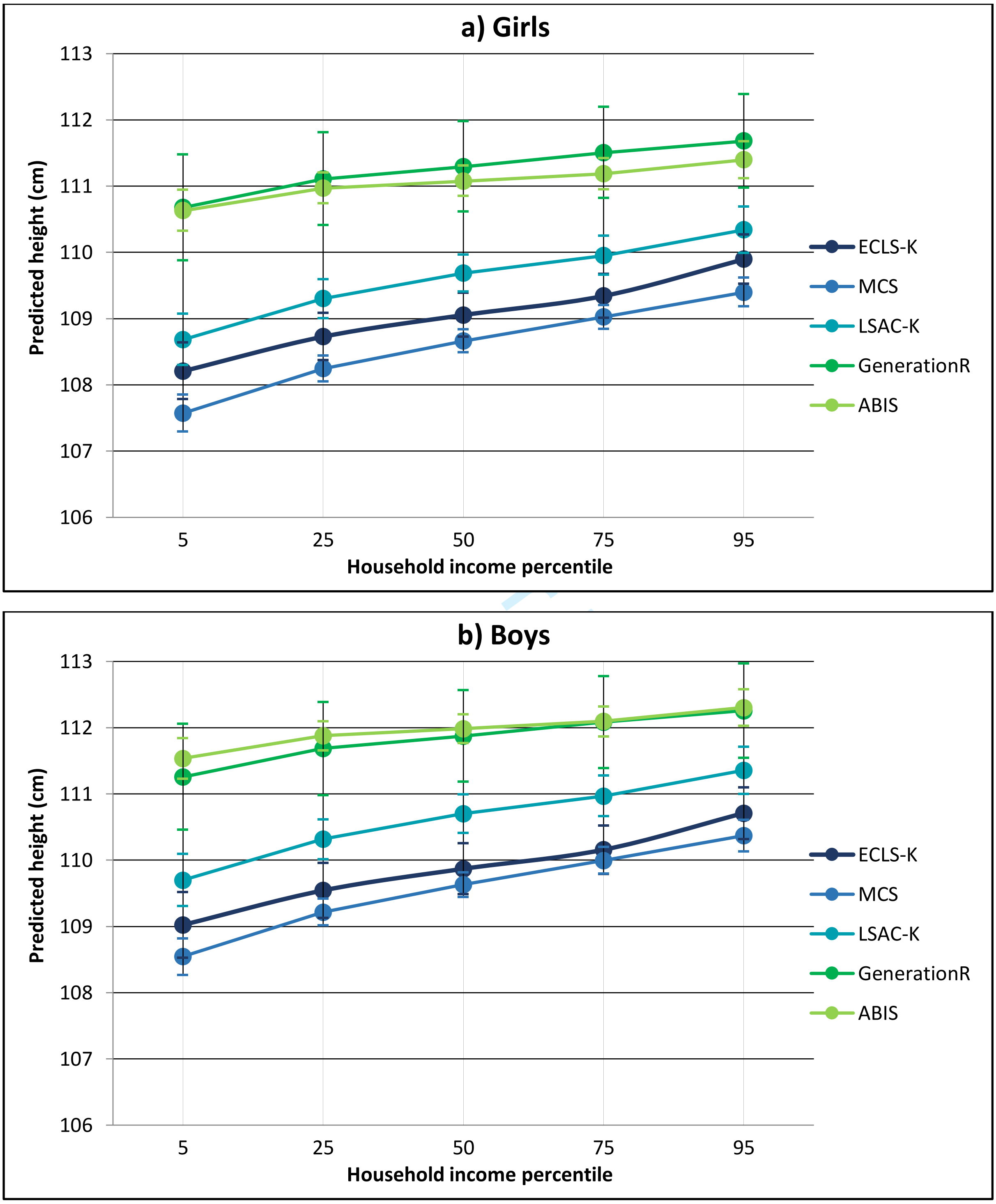

Figure 3: Layers of child health and development detriment

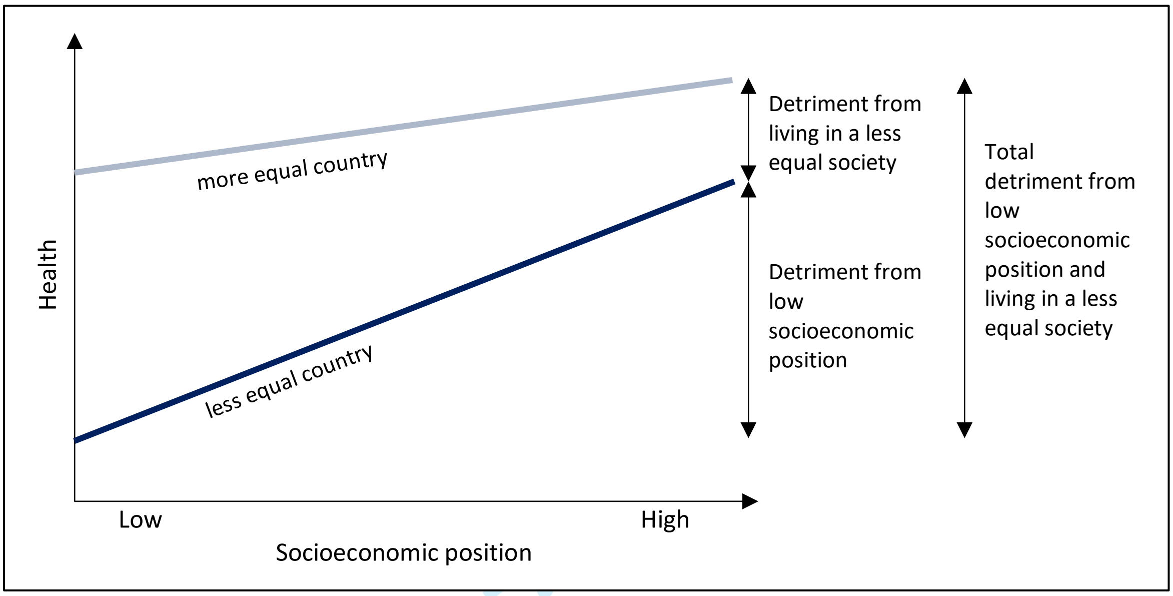

For Review Only

Income inequality and social gradients in children's height: a comparison of cohort studies from five high-income countries

Supplementary file

Supplementary Table 1a: Parental education response categories in each cohort and recoding strategies

**Harmonised parental education**	**ECLS-K (US)**	**MCS (UK)**	**LSAC-K (Australia)**
Level 1 (Below secondary school qualification/ not completed school)	8th grade or below<br>9th-12th grade	Academic qualification either of: CSE below grade 1/GCSE or O Level below grade C, SCE Standard, Ordinary grades below grade 3, or Junior Certificate below grade C<br>Or vocational qualification any of: NVQ or SVQ Level 1/GNVQ Foundation Level or GSVQ Level 1, BTEC, SCOTVEC first or general certificate/SCOTVEC modules, City & Guilds part 1/RSA Stage I,II,III/Junior certificate	Years of schooling <=10 AND highest qualification = has no certificate
Level 2 (Completed secondary school qualification)	High school diploma/ equivalent	Academic qualification either of: A/AS/S Levels/SCE Higher, Scottish Certificate Sixth Year Studies, Leaving Certificate or equivalent, Level or GCSE grade A-C, SCE Standard, Ordinary grades 1-3, or Junior Certificate grade A-C<br>Or vocational qualification any of: NVQ or SVQ Level 3/GNVQ Advanced or GSVQ Level 3, OND, ONCM BTEC National, SCOTVEC National Certificate, City & Guilds advanced craft, Part III/RSA Advanced Diploma, NVQ or SVQ Level 2/GNVQ Intermediate or GSVQ Level 2, BTEC, SCOTVEC first or general diploma, City & Guilds Craft or Part II/RSA Diploma	Years of schooling >=10 AND highest qualification = has a certificate
Level 3 (Post-secondary or technical qualification)	Vocational/ technical programme<br>Some college<br>Graduate/ professional school – no degree	Academic qualification either of: Post-graduate Diplomas and Certificates, Diplomas in higher education and other higher education qualifications, Teaching qualifications for schools or further education (below degree level)<br>Or vocational qualification any of: Nursing or other medical qualifications (below degree level), NVQ or SVQ level 4 or 5, HND, HNC, Higher Level BTEC/RSA Higher Diploma	Has trade qualification or highest qualification = advanced diploma/diploma
Level 4 (Degree or higher qualification)	Bachelor's degree<br>Masters degree<br>Doctorate/professional degree	Academic qualification either of: Higher Degree and Postgraduate qualifications, First Degree (including B.Ed.)<br>Or vocational qualification any of: Professional qualifications at degree level e.g. graduate member of professional institute, chartered	Highest qualification = bachelor degree or graduate diploma/certificate or postgraduate degree

Confidential: For Review Only

		accountant or surveyor	

Supplementary Table 1b: Parental education response categories in each cohort and recoding strategies

Harmonised parental education variable	GenerationR (Netherlands)	ABIS (Sweden)
Level 1(Completed secondary school qualification)	No primary school completed<br>Primary school completed (elementary education)<br>Special/remedial primary education (ZMOK, MLK, ZMLK, BLO, LOM)<br>Special secondary education (VSO-LOM, VSO-MLK)<br>Pre-vocational education (VBO)	Grundskola (elementary/ secondary school)
Level 2 (Lower technical/vocational qualification)	Lower vocational education / secondary general education (VMBO, MAVO, MULO)<br>General secondary education (HAVO, VWO, HBS, MMS, lyceum, atheneum, gymnasium)	Gymnasium, praktisk linje (senior high school – practical)
Level 3 (Higher technical/theoretical qualification)	Senior secondary vocational education (MBO, apprenticeship training)<br>Higher professional education (HBO)	Gymnasium, teoretisk linje (senior high school – theoretical)<br>Folkhögskola (residential college)
Level 4 (Degree or higher qualification)	University degree (WO)	Högskolautbildning högskole/univ-utbildning (college/university)

Supplementary Table 2a: Unadjusted gradients in child height by highest parental education level

	ECLS-K (US)		MCS (UK)		LSAC-K (Australia)		GenerationR (Netherlands)		ABIS (Sweden)	
	n	mean	n	mean	n	mean	n	mean	n	mean
**Girls**										
Level 1 (lowest)	127	111.7	876	109.1	147	107.6	74	118.2	58	112.5
Level 2	974	112.6	2498	109.9	502	107.7	371	118.4	606	113.5
Level 3	1721	113.1	660	110.0	663	108.1	456	118.4	1053	113.3
Level 4 (highest)	1697	113.3	1912	110.7	741	108.4	869	118.7	1585	113.8
Total	4519	113.0	5946	110.1	2053	108.0	1770	118.5	3302	113.6
**Boys**										
Level 1 (lowest)	159	113.4	873	110.0	129	108.6	94	119.7	77	113.5
Level 2	1042	113.6	2688	110.7	525	108.9	367	119.3	705	114.4
Level 3	1720	114.1	683	111.2	705	108.9	451	119.0	1154	114.5
Level 4 (highest)	1843	114.6	1992	111.8	779	109.3	830	119.3	1726	114.6
Total	4764	114.2	6236	111.0	2138	109.0	1742	119.2	3662	114.5

Note: Numbers are unweighted, means are weighted (unweighted in GenerationR and ABIS).

Confidential: For Review Only

Supplementary Table 2b: Unadjusted gradients in child height by equivalised household income quintile

	ECLS-K (US)		MCS (UK)		LSAC-K (Australia)		GenerationR (Netherlands)		ABIS (Sweden)	
**Household income quintile**	n	mean	n	mean	n	mean	n	mean	n	mean
**Girls**										
1 (lowest)	836	112.4	1261	109.4	358	107.3	317	118.4	625	113.0
2	883	112.9	1259	109.6	386	107.6	340	118.6	658	113.5
3	905	113.4	1181	110.1	386	108.0	327	119.0	680	113.3
4	942	113.2	1150	110.3	402	108.7	342	118.2	680	113.5
5 (highest)	953	113.2	1089	110.8	462	108.5	334	118.7	665	114.3
Total	4519	113.0	5940	110.1	1994	108.0	1660	118.6	3308	113.6
**Boys**										
1 (lowest)	845	113.4	1296	110.2	380	108.5	345	119.2	762	114.2
2	947	114.2	1324	110.6	401	108.6	322	118.9	740	114.4
3	959	114.3	1268	111.0	430	108.9	336	119.2	716	114.6
4	1000	114.3	1213	111.4	427	109.1	321	119.4	712	114.4
5 (highest)	1013	114.6	1129	111.8	441	109.8	327	119.4	724	114.9
Total	4764	114.2	6230	111.0	2079	109.0	1651	119.2	3654	114.5

Note: Numbers are unweighted, means are weighted (unweighted in GenerationR).

Confidential: For Review Only
